# Growth factor for therapeutic angiogenesis in ischemic heart disease: A meta-analysis of randomized controlled trials

**DOI:** 10.3389/fcell.2022.1095623

**Published:** 2022-12-09

**Authors:** Ling Tan, Lin-Zi Long, Hong-Zheng Li, Wen-Wen Yang, Yu-Xuan Peng, Jie-Ming Lu, Fei-Fei Liao, Xiao-Chang Ma, Hua Qu, Chang-Geng Fu, Shan-Shan Zhang

**Affiliations:** ^1^ Xiyuan Hospital, China Academy of Chinese Medical Sciences, Beijing, China; ^2^ Graduate School of Beijing University of Chinese Medicine, Beijing, China; ^3^ National Cardiovascular Clinical Medical Research Center of TCM, Beijing, China; ^4^ Beijing Xibeiwang Town Community Health Service Center, Beijing, China

**Keywords:** growth factor, therapeutic angiogenesis, ischemic heart disease, meta-analysis, randomized controlled study

## Abstract

**Aim:** This study was designed to systematically evaluate the effects of growth factor (GF) for therapeutic angiogenesis on ischemic heart disease (IHD) by pooling the results of randomized controlled trials (RCTs).

**Methods and Results:** PubMed, EMBASE, and CENTRAL databases were searched from inception to October 2022. RCTs, investigating the effects of GF therapy on IHD, were included. The risk bias of included study was assessed according to Cochrane tool. Weighted mean difference (WMD), calculated with fixed effect model or random effect model, was used to evaluate the effects of GF therapy on left ventricular ejection fraction (LVEF) and Canadian Cardiovascular Society (CCS) angina class. Relative risk (RR) was used to evaluate the effects of GF therapy on all-cause mortality, major adverse cardiovascular events (MACE) and revascularization. Meta-analysis, meta-regression analysis and publication bias analysis were performed by RevMan 5.3 or Stata 15.1 software. Twenty-nine studies involving 2899 IHD patients (1,577 patients in GF group and 1,322 patients in control group) were included. Compared with the control group, GF therapy did not reduce all-cause mortality (RR: 0.82; 95% CI: 0.54–1.24; *p* = 0.341), MACE [(RR: 0.83; 95% CI: 0.61–1.12; *p* = 0.227), revascularization (RR: 1.27, 95% CI: 0.82–1.96, *p* = 0.290) and CCS angina class (WMD: −0.08, 95% CI: −0.36 to 0.20, *p* = 0.560). However, GF therapy could increase LVEF during short-term follow-up (<1 year).

**Conclusion:** GF for therapeutic angiogenesis was beneficial for increasing LVEF during short-term follow-up (<1 year), however, the therapy was not efficacious in decreasing all-cause mortality, MACE and revascularization.

## 1 Introduction

Ischemic heart disease (IHD) is the major cause of death all over the world according to the report of World Health Organization (WHO) (Roth et al., 2020). Even though guideline-based medical therapy and percutaneous coronary intervention (PCI) have been widely used in IHD patients, the 4-year rate of death and myocardial infarction of the patients remain about 10% (Maron et al., 2020). Lots of patients could not benefit from PCI (Ng et al., 2022), and some patients also suffer from refractory angina despite intensive medical therapy (Park and Conti, 2018).

IHD is characterized by decreased coronary blood flow, and increasing blood flow in the area of ischemic myocardium is main therapeutic aim (Heusch, 2016). Therapeutic angiogenesis, including vascular endothelial growth factor (VEGF), placental growth factor (PLGF), fibroblast growth factor (FGF), hepatocyte growth factor (HGF), platelet-derived growth factor (PDGF), angiopoietin (Ang) and erythropoietin (EPO), might be novel treatment options for IHD patients. Previous animal study demonstrated that therapeutic angiogenesis could increase blood flow and local blood vessel numbers in the area of ischemia (Shams et al., 2022). However, the results are controversial in clinical trials. Voors et al. (2010) found that GF for therapeutic angiogenesis represented by EPO was related to less major adverse cardiovascular events (MACE). Nevertheless, the study performed by Steppich et al. (2017) showed that GF for therapeutic angiogenesis did not promote target vessel revascularization in patients with IHD compared with placebo. Therefore, the present meta-analysis of randomized controlled trials (RCTs) was designed to assess the effect of therapeutic angiogenesis on IHD patients.

## 2 Methods

### 2.1 Data source and searches

The meta-analysis was performed according to the Preferred Reporting Items for Systematic Reviews and Meta-Analyses (PRISMA) guidelines (Liberati et al., 2009), and the protocol was registered in INPLASY (No.2022110041, https://inplasy.com/). Two investigators (TL and ZSS) independently performed the database search and study selection. PubMed, EMBASE, and CENTRAL database were searched with the terms: (“vascular endothelial growth factor” or “placental growth factor” or “fibroblast growth factor” “hepatocyte growth factor” or “platelet-derived growth factor” or “angiopoietin” or “erythropoietin”) and (“ischemic heart disease” or “coronary artery disease” or “coronary heart disease”) and (“randomized controlled trial” or “clinical trial”) from inception to October 2022. The detailed search strategies are listed in Supplementary Table S1. We also performed a manual search according to associated published review.

### 2.2 Study selection

Studies were included if they met the following inclusion criteria: 1) RCT studies comparing GF therapy and standard treatments for IHD; 2) the participants have myocardial hypoperfusion according to perfusion imaging, and have been diagnosed as acute coronary syndrome or chronic ischemic heart disease (Buja and Vander Heide, 2016); 3) reported the outcome including all-cause mortality, MACE, revascularization, left ventricular ejection fraction (LVEF), or Canadian Cardiovascular Society (CCS) angina class at least one. The studies were excluded if: 1) the data of outcome was not available 2) the studies were published as comments, conference abstracts, or letters to the editor.

### 2.3 Data extraction and quality assessment

Two investigators (TL and ZSS) extracted data from included studies independently. The disagreements would be resolved by consulting a third investigator (FCG). The following study characteristics were collected: first author, publication year, follow-up duration, type of IHD, categories of growth factors for interventions, control, sample size, age at entry, percentage of male participants, and key outcomes.

The primary outcomes of this study were all-cause mortality and MACE, and the second outcomes were revascularization, LVEF and CCS angina class. When the data of outcome was unavailable, we will try to connect the corresponding author. Two investigators (TL and ZSS) assessed the risk of bias of the included studies with the Cochrane tool. The categorization of “low risk,” “high risk,” or “some concerns” was applied to the included studies according to the following domains: random sequence generation, allocation concealment, blinding of participants and personnel, blinding for outcome assessment, incomplete outcome data, selective reporting, and other potential sources of bias. Disagreements were resolved through discussing with a third investigator (FCG).

### 2.4 Statistical analysis

Dichotomous outcomes were analyzed with the relative risk (RR) and 95% confidence interval (CI). Continuous outcomes were analyzed with the weighted mean difference (WMD) and 95% CI. I^2^ statistic was used to measure the heterogeneity among the studies. When I^2^ ≤ 50%, we will consider that the heterogeneity was not significant among the studies, and fixed-effect models will be used. I^2^ > 50% indicated that the heterogeneity among the studies was statistically significant, random-effect models will be applied. Meta-regression analysis was conducted for the factors that affected the research results, such as the baseline LVEF values and baseline CCS angina class, to observe their impact on outcomes. If necessary, subgroup analysis based on factors such as type of IHD, categories of growth factors, injection methods and follow-up duration was conducted to clarify their impact on outcome. Sensitivity analysis was used to observe whether the results were reliable after the studies were excluded one by one. Publication bias was evaluated using funnel plots and egger’s test. The data were analyzed with Stata (version 15.1) or Cochrane Collaboration software (RevMan 5.3).

## 3 Results

### 3.1 Study selection

**FIGURE 1:**
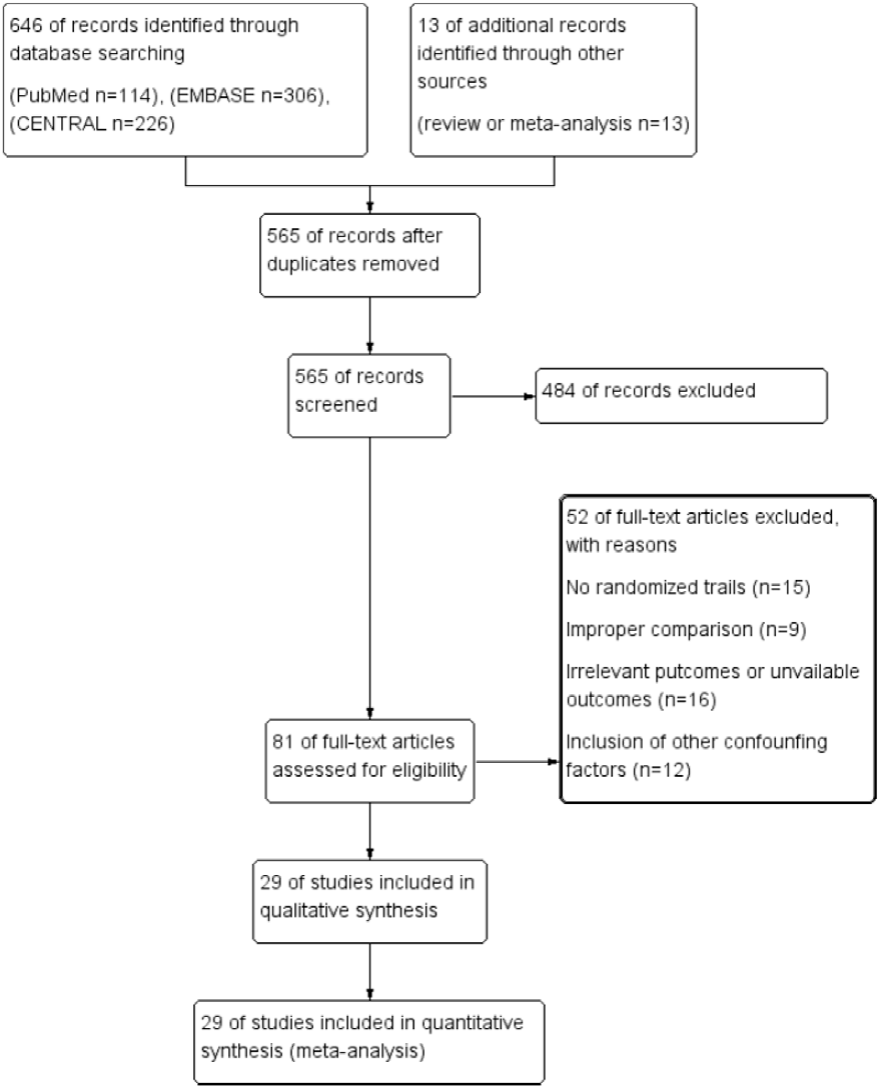
Flowchart of literature search and study selection.

The process of study selection was shown as Figure 1. Six hundred and fifty-nine articles (114 from PubMed, 306 from EMBASE, 226 from CENTRAL, and 13 additional records identified through literature review) were identified. Ninety-four articles were excluded for duplication, and remained 484 irrelevant articles were excluded after screening titles and abstracts. The remained 81 full-text articles were then assessed for eligibility, and 52 articles were excluded for no randomized controlled trials (*n* = 15), improper comparisons (*n* = 9), irrelevant outcomes and/or unavailable outcomes (*n* = 16) and inclusion of other confounding factors (*n* = 12). Finally, 29 articles were included in this meta-analysis (Voors et al., 2010; Steppich et al., 2017; Kukuła et al., 2019; Orii et al., 2018; Minamino et al., 2018; Meng et al., 2018; Ziabakhsh-Tabary et al., 2014; Fokkema et al., 2013; Prunier et al., 2012; Chih et al., 2012; Najjar et al., 2011; Kukuła et al., 2011; Ludman et al., 2011; Kastrup et al., 2011; Ozawa et al., 2010; Ott et al., 2010; Wang et al., 2009; Stewart et al., 2009; Hedman et al., 2009; Kastrup et al., 2005; Henry et al., 2003; Stewart et al., 2006).

### 3.2 Quality assessment

**FIGURE 2:**
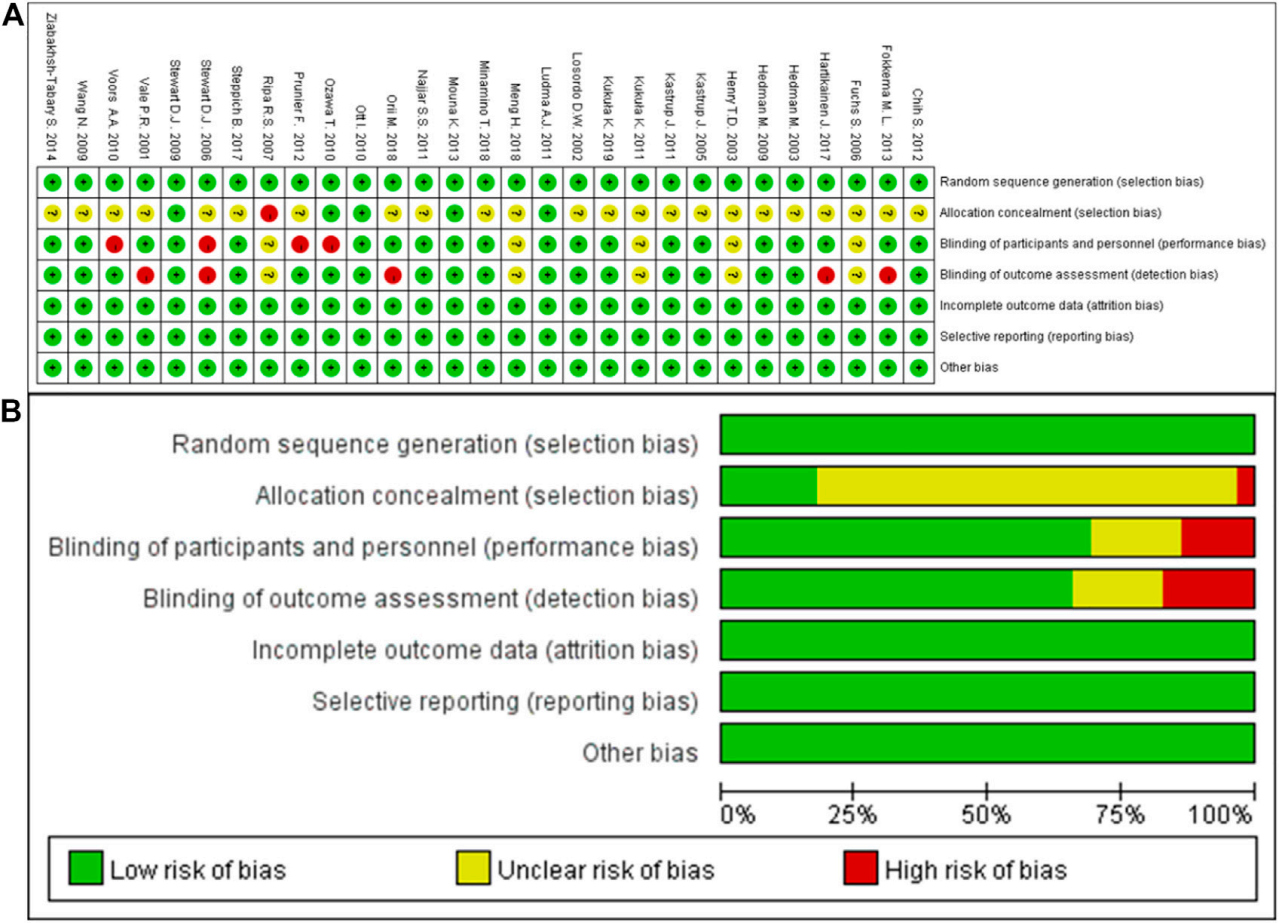
Risk of bias. **(A)**, Risk of bias summary: each risk of bias item for each included study; **(B)**, risk of bias graph: each risk of bias item presented as percentages across all included studies.

The quality of included studies were assessed based on seven aspects of risk biases, including random sequence generation, allocation concealment, blinding of participants and personnel, blinding for outcome assessment, incomplete outcome data, selective reporting, and other potential sources of bias. The results of quality assessment were shown as Figure 2. Overall, no attrition bias or reporting bias was observed, and the methods of random and blinding were considered to be adequate in this meta-analysis, but there was an unclear risk in allocation concealment.

### 3.3 Characteristics of included studies

The studies were published between 2001 and 2019. A total of 2899 IHD patients, aged from 56 to 72 years old, were included in the analysis. Six of the 29 studies contained 2 arms (Hedman et al., 2003; Henry et al., 2003; Ripa et al., 2006; Hedman et al., 2009; Minamino et al., 2018; Orii et al., 2018). The GFs involved VEGF, EPO, HGF, and Granulocyte Colony-Stimulating Factor (G-CSF), of which VEGF was subtyped into phVEGF, VEGF-A_165_, VEGF-A_121_, rhVEGF, and VEGF-D. Overall, 29 studies involving 2899 IHD patients (1,577 patients in GF group and 1,322 patients in control group) were included (Table 1).

**TABLE 1:** Basic characteristics of individuals.

Study	Duration	Type of IHD	Intervention	Control	Participants (T/C)	Age (y)		Men (%)		Outcome	Dosage
						Trial	Control	Trial	Control		
Kukuła et al. (2019)	133 months	Refractory CAD	VEGF-A_165_/bFGF plasmid (VIF)	placebo plasmid	52 (33/19)	—	—	—	—	All-cause mortality	—
Orii et al. (2018)	1 month	The first-time AMI	EPO	Saline	61 (32/29)	68 ± 12	71 ± 11	81.0	79.0	LVEF	12,000 IU
Orii et al. (2018)	8 months	The first-time AMI	EPO	Saline	61 (32/29)	68 ± 12	71 ± 11	81.0	79.0	LVEF	12,000 IU
Minamino et al. (2018)	6 months	The first-time STEMI	EPO	NA	128 (68/60)	61.3 ± 10.7	60.2 ± 10.7	85.3	86.7	LVEF, MACE, revascularization	12,000 IU
Minamino et al. (2018)	6 months	The first-time STEMI	EPO	NA	129 (69/60)	61.4 ± 11.6	60.2 ± 10.7	85.5	86.7	LVEF, MACE, revascularization	6,000I U
Meng et al. (2018)	6 months	Post-infarct heart failure	Ad-HGF	NA	30 (15/15)	63.1 ± 1.9	63.2 ± 2.2	100	80	LVEF	5 * 10^9^ pfu/ml, 1 * 10^10^ pfu/ml, or 2 * 10^10^ pfu/ml
Steppich et al. (2017)	6 months	The first-time STEMI	EPO	NA	138 (68/70)	59.1 ± 13.0	62.1 ± 12.3	82	74	All-cause mortality, MACE, revascularization	3.33 × 10^4^ IU
Ziabakhsh-Tabary et al. (2014)	1 month	Ischemia-Reperfusion Injuries	EPO	Saline	43 (22/21)	59.73 ± 7.73	62.57 ± 8.60	59.1	38.1	LVEF	700 IU/kg
Fokkema et al. (2013)	12 months	The first-time STEMI	EPO	NA	485 (236/249)	60.1 ± 10.5	60.5 ± 11.0	76.3	79.9	All-cause mortality, revascularization	60,000 IU
Prunier et al. (2012)	3 months	The first-time STEMI	EPO	NA	110 (57/53)	57.6 ± 11.7	57.3 ± 12.8	82	81	All-cause mortality, LVEF, revascularization	1,000 IU/kg
Chih et al. (2012)	12 weeks	SCHD	G-CSF	NA	36 (18/18)	62 ± 7	62 ± 7	89	89	LVEF	4.5 mg/kg/day
Najjar et al. (2011)	3 months	Acute STEMI	EPO	Saline	138 (68/70)	55.6 ± 12.6	57.4 ± 11.9	89.7	80	All-cause mortality, LVEF	6,000 IU
Kukuła et al. (2011)	12 months	Refractory CAD	VEGF/FGF plasmid	“empty” pSEC plasmid	52 (33/19)	62.86 ± 8.7	61.77 ± 8.07	72.7	84.2	CCS class, MACE, LVEF	0.5 mg
Ludman et al. (2011)	4 months	STEMI undergoing PPCI	EPO	Saline	51 (26/25)	55.5 ± 112.8	61 ± 10.0	88	84	All-cause mortality, revascularization	50,000 IU
Kastrup et al. (2011)	52 weeks	SCHD	Adenovirus carrying VEGF121	NA	17 (12/5)	60.9 ± 9.04	64.1 ± 7.38	75	80	LVEF, CCS class	4×10^10^ PU
Voors et al. (2010)	6 weeks	The first-time STEMI	EPO	NA	529 (263/266)	60.8 ± 10.9	61.0 ± 11.3	75.7	79.7	LVEF	60,000 IU
Ozawa et al. (2010)	6 months	Acute STEMI	EPO	Saline	36 (20/16)	59.4 ± 13.9	62.5 ± 8.0	87.5	80	LVEF	12,000 IU
Ott et al. (2010)	6 months	The first-time STEMI	EPO	NA	138 (68/70)	59.1 ± 13.0	62.1 ± 12.3	82	74	LVEF, revascularization, All-cause mortality	3.33 × 10^4^ IU
Hedman et al. (2009)	8 years	SCHD	VEGF-adenovirus	Ringer’s lactate	75 (37/38)	58 ± 8	56 ± 9	70.3	78.9	All-cause mortality, MACE, CCS class	2000 μl
Hedman et al. (2009)	8 years	SCHD	VEGF-plasmid/liposome	Ringer’s lactate	66 (28/38)	58 ± 7	56 ± 9	82.1	78.9	All-cause mortality, MACE, CCS class	2000 μl
Wang et al. (2009)	2 weeks	Severe CAD	pHGF	Saline	49 (21/28)	72.42 ± 11.82	67.86 ± 10.67	71.4	71.4	LVEF	2 mg/kg
Kastrup et al. (2005)	3 months	SCHD	phVEGF-A_165_	Placebo plasmid	80 (40/40)	61 ± 2	61 ± 2	52.5	57.4	LVEF, CCS class	0.5 mg
Henry et al. (2003)	120 days	SCHD	rhVEGF	NA	119 (56/63)	61 ± 9	61 ± 7	89	87	All-cause mortality, CCS class, LVEF, revascularization	17 ng kg^−1^·min^−1^
Henry et al. (2003)	120 days	SCHD	rhVEGF	NA	122 (59/63)	58 ± 8	61 ± 7	92	87	All-cause mortality, CCS class, LVEF, revascularization	50 ng kg^−1^·min^−2^
Hartikainen et al. (2017)	12 months	Refractory CAD	VEGF-D^ΔNΔC^	0.9% NaCl	30 (24/6)	71 ± 6	70 ± 6	96	83	CCS class, All-cause mortality, MACE	2 ml
Muona et al. (2013)	3 months	SCHD	VEGF-D^ΔNΔC^	NA	15 (12/3)	71.25 ± 5.12	68.33 ± 3.79	100	100	All-cause mortality	2 ml
Stewart et al. (2009)	6 months	Advanced Coronary	VEGF_165_	Buffered saline	93 (48/45)	63 ± 7	64 ± 8	83	93	All-cause mortality	2 ml
Stewart et al. (2006)	12 months	SCHD	AdVEGF_121_	Maximum medical treatment	67 (32/35)	61	60	84	94	All-cause mortality	4×10^10^ p.u
Ripa et al. (2006)	3 months	SCHD	VEGF-A_165_ and G-CSF	Placebo plasmid	32 (16/16)	62 ± 9	62 ± 9	87.5	87.5	LVEF, CCS class	0.5 mg
Ripa et al. (2006)	3 months	SCHD	VEGF-A_165_	Placebo plasmid	32 (16/16)	61 ± 7	62 ± 9	93.8	87.5	LVEF, CCS class	0.5 mg
Fuchs et al. (2006)	52 weeks	Refractory CAD	AdVEGF121	Diluent	10 (6/4)	61	69	83	100	LVEF, CCS class	4×10^10^ p.u
Hedman et al. (2003)	6 months	SCHD	VEGF-Adv	Ringer’s lactate	75 (37/38)	58 ± 8	56 ± 9	70.3	78.9	All-cause mortality, MACE	2 × 10^10^ pfu
Hedman et al. (2003)	6 months, 28 months	SCHD	VEGF-P/L	Ringer’s lactate	66 (28/38)	58 ± 7	56 ± 9	82.1	78.9	All-cause mortality, MACE	2000 μg
Losordo et al. (2002)	12 weeks	SCHD	phVEGF-2	Saline	19 (12/7)	62 ± 3	59 ± 3	75	85.7	LVEF, CCS class	6.0 ml
Vale et al. (2001)	3 months, 12 months	Refractory CAD	phVEGF-2	Mock procedure	9 (6/3)	—	—	—	—	LVEF	200 μg

Abbreviations: T, trial; C, control; IHD, ischemic heart disease; CAD, coronary heart disease, STEMI, ST, segment elevation myocardial infarction; SCHD, stable coronary heart disease; MACE, major adverse cardiovascular events; PPCI, primary percutaneous coronary intervention; LVEF, left ventricular ejection fraction; CCS, canadian cardiovascular society; VEGF, vascular endothelial growth factor; EPO, erythropoietin; HGF, hepatocyte growth factor; G-CSF, granulocyte colony-stimulating factor.

### 3.4 Primary outcomes

#### 3.4.1 All-cause mortality

**FIGURE 3:**
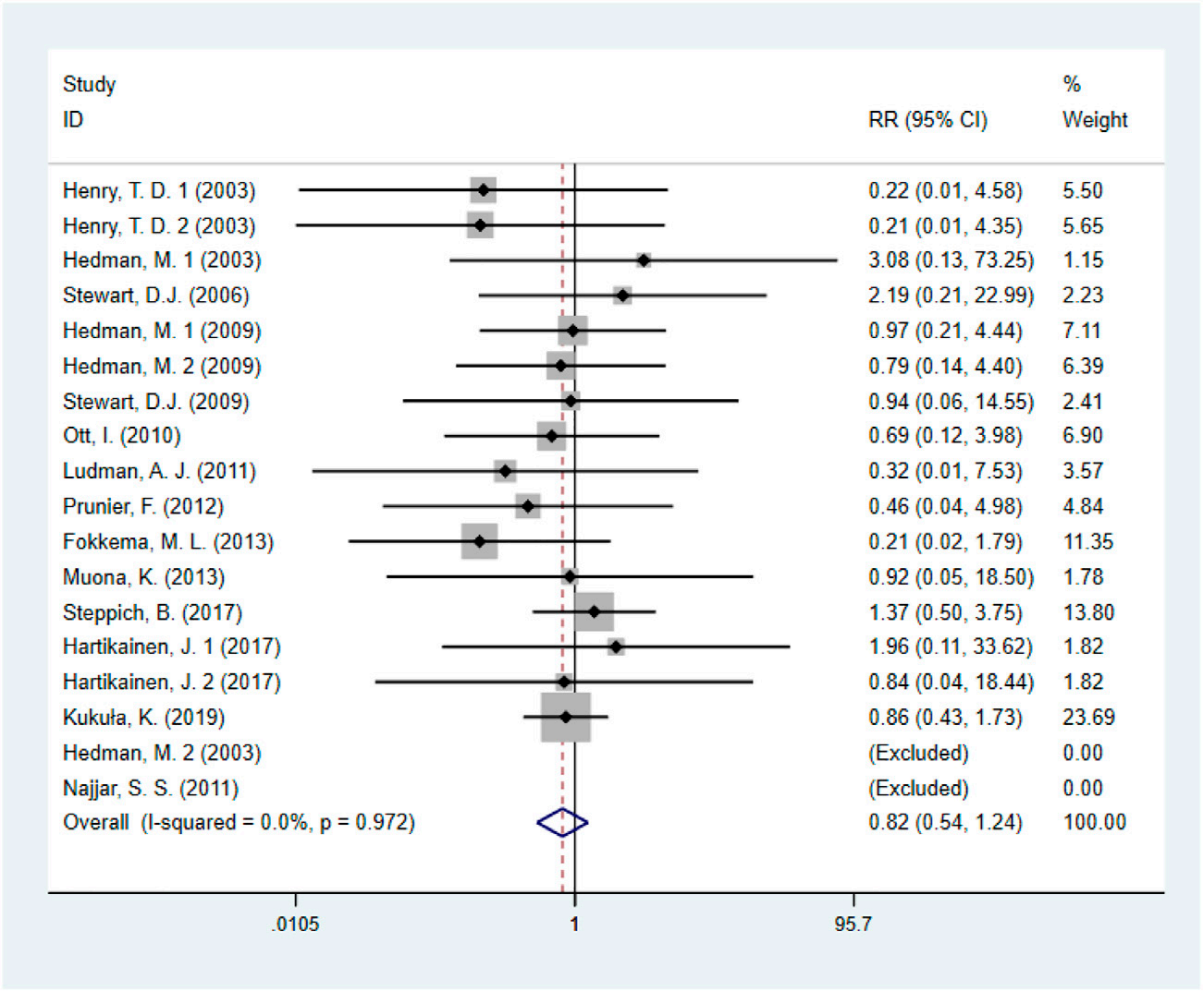
Forest plot for all-cause mortality, GF vs. control. RR, relative risk; CI, confidence interval; ID, identification.

Fourteen studies (Steppich et al., 2017; Kukuła et al., 2019; Fokkema et al., 2013; Prunier et al., 2012; Najjar et al., 2011; Ludman et al., 2011; Ott et al., 2010; Stewart et al., 2009; Hedman et al., 2009; Henry et al., 2003; Hedman et al., 2003; Hartikainen et al., 2017), including 1872 IHD patients (952 patients in GF group vs. 920 patients in control group), evaluated the effects of GF on all-cause mortality. The results demonstrated that there was no statistical difference between the GF therapy group and the control group in decreasing all-cause mortality (RR: 0.82; 95% CI: 0.54–1.24; *p* = 0.341) (Figure 3). And no obvious heterogeneity between studies was observed (*I* ^*2*^ = 0.0%, *p* = 0.972). Results from sensitivity analysis showed that exclusion of any single study did not affect the overall estimate for the effects of GF on all-cause mortality. We also performed subgroup analysis based on type of IHD, categories of growth factors, injection methods and follow-up duration, and the results showed that those factors did not influence the final effect size (Supplementary Figures S1–S4; Table 2).

**TABLE 2:** Subgroup analyses of growth factors on IHD.

Variable	NO.	WMD (95%CI)	I^2^ (%)	P Heterogeneity	P Within group
Subgroups analyses of growth factors on all-cause mortality					
Overall effect	18	0.82 (0.54, 1.24)	0	0.972	0.341
Type of IHD					
Refractory CAD	4	0.94 (0.49, 1.80)	0	0.957	0.841
STEMI	6	0.73 (0.36, 1.47)	0	0.522	0.377
SCHD	8	0.82 (0.37, 1.82)	0	0.826	0.626
Categories of growth factors					
VEGF	12	0.88 (0.53, 1.46)	0	0.978	0.618
EPO	6	0.73 (0.36, 1.47)	0	0.522	0.377
Injection methods					
Intramyocardial injections	3	0.97 (0.51, 1.87)	0	0.752	0.937
Intravenous injection	6	0.73 (0.36, 1.47)	0	0.522	0.377
Intracoronary infusion	4	0.51 (0.13, 1.96)	0	0.667	0.328
Gene transfer	5	1.04 (0.39, 2.81)	0	0.905	0.932
Follow-up duration					
Long term (≥1 year)	8	0.82 (0.48, 1.41)	0	0.852	0.479
Short term (<1 year)	10	0.81 (0.43, 1.54)	0	0.876	0.523
Subgroups analyses of growth factors on MACE					
Overall effect	7	0.83 (0.61, 1.12)	13.4	0.328	0.227
Type of IHD					
Refractory CAD	1	2.00 (0.31, 13.06)	—	—	0.469
STEMI	4	0.83 (0.54, 1.29)	44.7	0.143	0.418
SCHD	2	0.75 (0.49, 1.15)	0	0.486	0.193
Categories of growth factors					
VEGF	3	0.82 (0.54, 1.24)	0	0.480	0.348
EPO	4	0.83 (0.54, 1.29)	44.7	0.143	0.418
Injection methods					
Intravenous injection	4	0.83 (0.54, 1.29)	44.7	0.143	0.418
Intramyocardial injections	1	2.00 (0.31, 13.06)	—	—	0.469
Gene transfer	2	0.75 (0.49, 1.15)	0	0.486	0.193
Follow-up duration					
Long term (≥1 year)	2	0.75 (0.49, 1.15)	0	0.486	0.193
Short term (<1 year)	5	0.88 (0.58, 1.34)	34.3	0.193	0.555
Subgroups analyses of growth factors on revascularization					
Overall effect	10	1.27 (0.82, 1.96)	0	0.730	0.290
Type of IHD					
Refractory CAD	1	—	—	—	—
STEMI	7	1.28 (0.81, 2.00)	0	0.514	0.285
SCHD	2	1.10 (0.16, 7.65)	0	0.979	0.926
Categories of growth factors					
VEGF	3	1.10 (0.16, 7.65)	0	0.979	0.926
EPO	7	1.28 (0.81, 2.00)	0	0.514	0.285
Injection methods					
Intravenous injection	7	1.28 (0.81, 2.00)	0	0.514	0.285
Intramyocardial injections	1	—	—	—	—
Intracoronary infusion	2	1.10 (0.16, 7.65)	2	0.979	0.926
Follow-up duration					
Long term (≥1 year)	2	0.81 (0.36, 1.82)	—	—	0.611
Short term (<1 year)	8	1.55 (0.91, 2.64)	0	0.837	0.103
Subgroups analyses of growth factors on LVEF					
Overall effect	25	2.05 (1.64, 2.46)	11.4	0.301	<0.001
Type of IHD					
Refractory CAD	11	0.70 (−1.41, 2.82)	0	0.881	0.514
STEMI	8	0.78 (−0.60, 2.16)	0	0.792	0.267
SCHD	5	2.88 (2.27, 3.50)	3.5	0.387	<0.001
MIHF	1	1.60 (0.99, 2.21)	—	—	<0.001
Categories of growth factors					
VEGF	11	2.80 (2.20, 3.41)	5.4	0.392	<0.001
EPO	11	0.80 (−0.48, 2.09)	0	0.934	0.222
G-CSF	1	1.00 (−7.84, 9.84)	—	—	0.824
pHGF	2	1.58 (0.97, 2.19)	0	0.571	<0.001
Injection methods					
Intravenous injection	13	0.70 (−0.47, 1.86)	0	0.982	0.241
Intramyocardial injections	7	1.21 (−2.52, 4.93)	1.6	0.412	0.525
Subcutaneous injection	1	1.00 (−7.84, 9.84)	—	—	0.824
Gene transfer	3	2.95 (2.32, 3.58)	0	0.483	<0.001
Percutaneous endocardial injection	1	1.60 (0.99, 2.21)	—	—	<0.001
Follow-up duration					
Long term (≥1 year)	6	1.99 (−1.30, 5.29)	0	0.570	0.236
Short term (<1 year)	19	2.05 (1.64, 2.46)	22.5	0.182	<0.001
Baseline LVEF (%)					
<50	12	1.42 (0.88, 1.97)	0	0.865	<0.001
≥50	13	2.81 (2.21, 3.42)	0	0.630	<0.001
Subgroups analyses of growth factors on CCS class					
Overall effect	10	−0.08 (−0.36, 0.20)	96.5	<0.001	0.560
Type of IHD					
Refractory CAD	2	0.02 (−0.37, 0.41)	0	0.708	0.918
SCHD	8	−0.11 (−0.42, 0.20)	97.3	<0.001	0.502
Injection methods					
Intramyocardial injections	6	−0.17 (−0.56, 0.21)	98	<0.001	0.375
Intracoronary infusion	4	0.05 (−0.13, 0.23)	0	0.942	0.619
Follow-up duration					
Long term (≥1 year)	5	−0.05 (−0.36, 0.25)	0	0.668	0.724
Short term (<1 year)	5	−0.08 (−0.44, 0.27)	98.4	<0.001	0.644
Baseline CCS angina class					
≥3.0	4	0.05 (−0.13, 0.23)	0	0.942	0.619
<3.0	6	−0.17 (−0.56, 0.21)	98	<0.001	0.375

Abbreviations: CI, confidence interval; IHD, ischemic heart disease; CAD, coronary heart disease, STEMI, ST, segment elevation myocardial infarction; SCHD, stable coronary heart disease; MIHF, heart failure after myocardial infarction; VEGF, vascular endothelial growth factor; EPO, erythropoietin; HGF, hepatocyte growth factor; G-CSF, granulocyte colony-stimulating factor.

#### 3.4.2 Major adverse cardiovascular events

**FIGURE 4:**
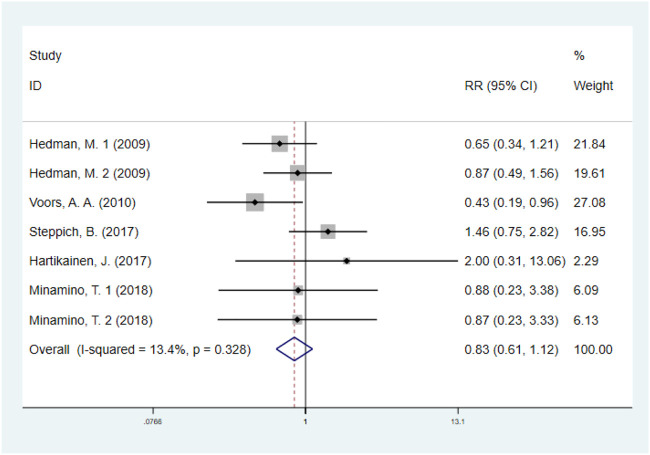
Forest plot for MACE, GF vs. control. MACE, major adverse cardiovascular events; RR, relative risk; CI, confidence interval; ID, identification.

The effect of GF on MACE was evaluated in five studies with 1074 IHD patients (Hedman et al., 2009; Voors et al., 2010; Hartikainen et al., 2017; Steppich et al., 2017; Minamino et al., 2018) (550 patients in GF group vs. 524 patients in control group). The results showed that GF therapy did not significantly decrease the risk of MACE compared to the control group (RR: 0.83; 95% CI: 0.61–1.12; *p* = 0.227). In addition, low significant heterogeneity was observed regarding this outcome (*I* ^*2*^ = 13.4%, *p* = 0.328) (Figure 4). Subgroup analyses based on type of IHD, categories of growth factors, injection methods and follow-up duration were performed, and the final results were not influenced (Supplementary Figures S5–S8; Table 2). Sensitivity analysis showed that deletion of any one study did not alter the overall estimate for the impact of GF on MACE.

### 3.5 Secondary outcomes

#### 3.5.1 Revascularization

**FIGURE 5:**
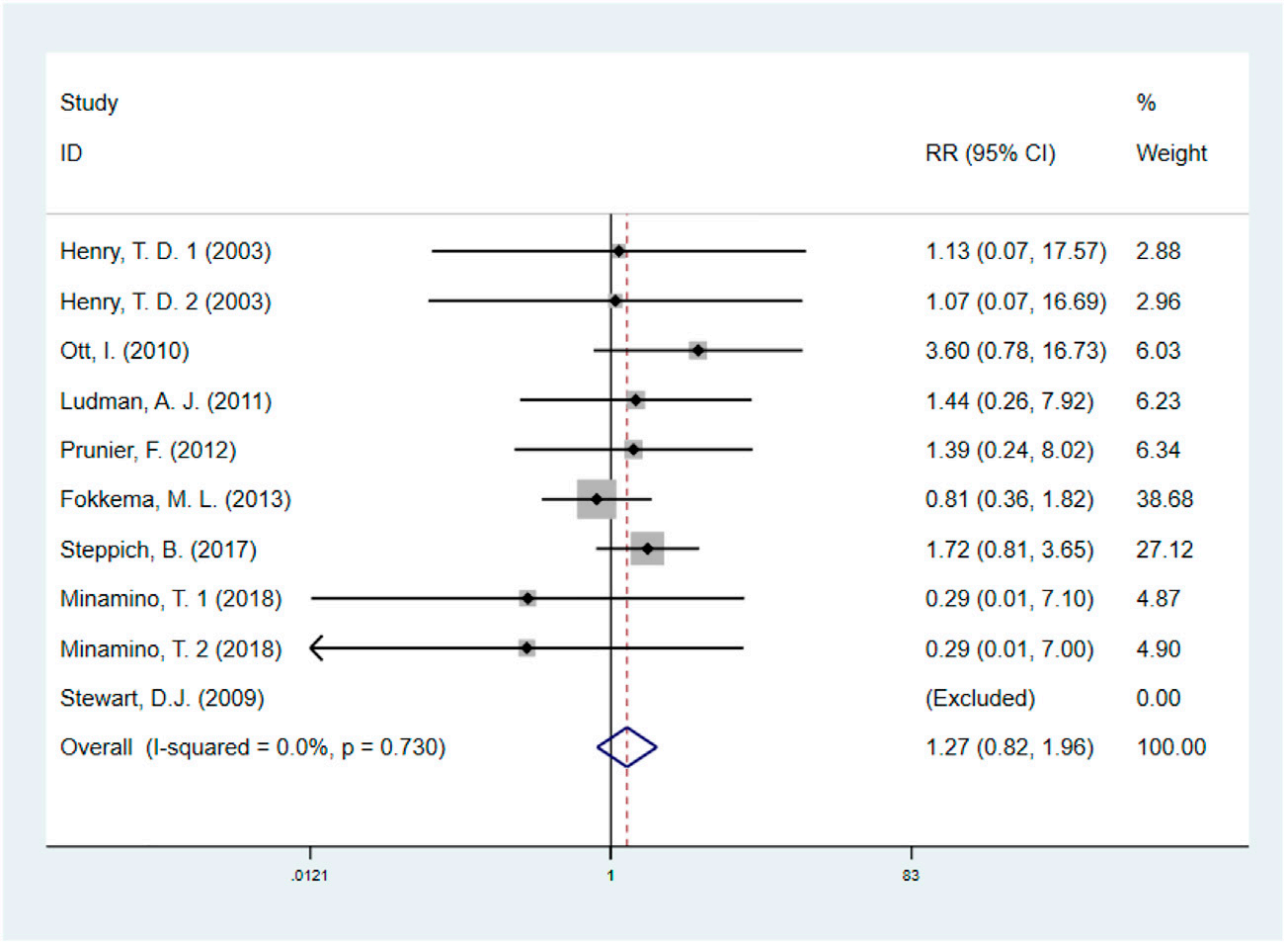
Forest plot for revascularization, GF vs. control. RR, relative risk; CI, confidence interval; ID, identification.

A total of eight studies (Henry et al., 2003; Stewart et al., 2009; Ott et al., 2010; Ludman et al., 2011; Prunier et al., 2012; Fokkema et al., 2013; Steppich et al., 2017; Minamino et al., 2018), including 1513 IHD patients (755 patients in GF group vs. 758 patients in control group), investigated the effects of GF therapy on revascularization. Pooled effect sizes from the eligible studies indicated that there was no significant difference on revascularization between the GF group and control group (RR: 1.27, 95% CI: 0.82–1.96, *p* = 0.290) (Figure 5). And no obvious heterogeneity was found regarding the outcome (I^2^ = 0.0%, *p* = 0.730). Subgroup analyses were performed by type of IHD, categories of GF, injection methods, and follow-up duration, and the results were unchanged, indicating that GF does not increase revascularization in IHD patients (*p* > 0.05) (Supplementary Figures S9–S12; Table 2). The sensitivity analysis showed that exclusion of any single study did not affect the overall estimate for the effect of GF on revascularization.

#### 3.5.2 Left ventricular ejection fraction

**FIGURE 6:**
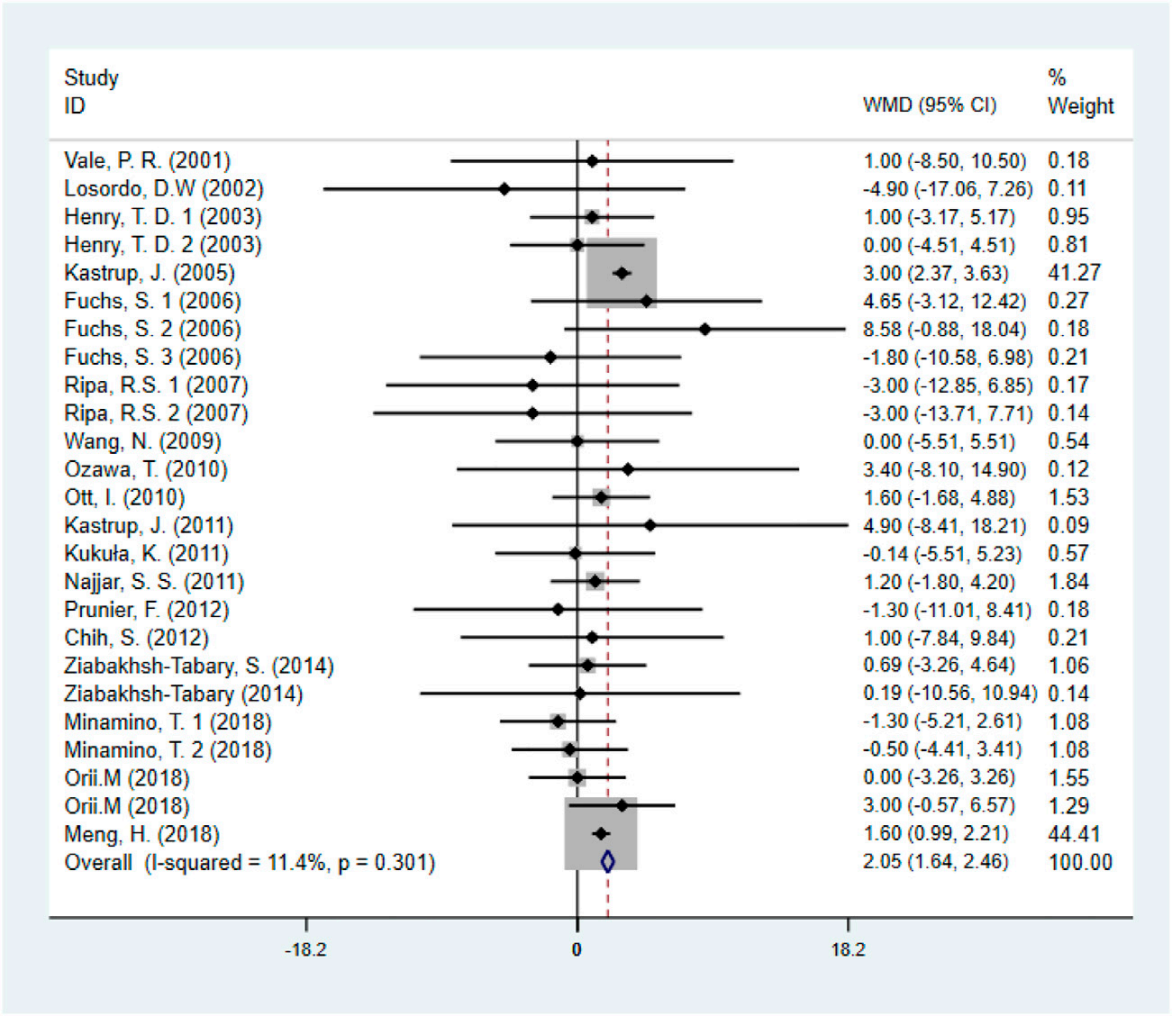
Forest plot for LVEF, GF vs. control. LVEF, left ventricular ejection fraction; RR, relative risk; CI, confidence interval; ID, identification.

**FIGURE 7:**
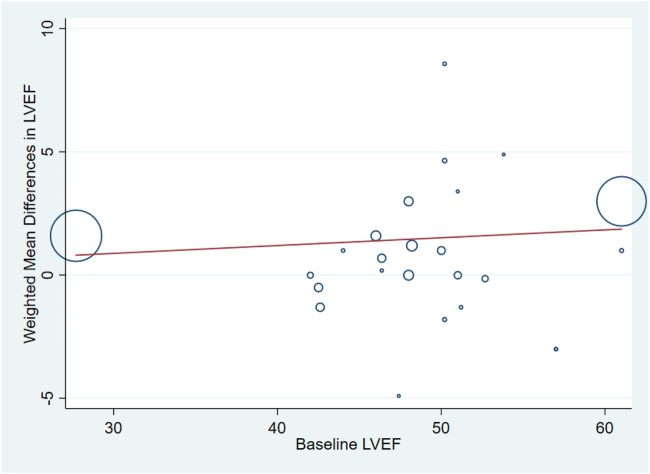
Meta-regression plots of the weighted mean difference in LVEF according to baseline LVEF (*p* = 0.429). LVEF, left ventricular ejection fraction.

Eighteen studies (Minamino et al., 2018; Ziabakhsh-Tabary et al., 2014; Prunier et al., 2012; Chih et al., 2012; Najjar et al., 2011; Kukuła et al., 2011; Kastrup et al., 2011; Ozawa et al., 2010; Ott et al., 2010; Wang et al., 2009; Kastrup et al., 2005; Henry et al., 2003), including 1,446 patients (750 individuals in GF group vs. 696 individuals in control group) assessed the effect of GF on LVEF. Pooled results showed that GF therapy led to a significantly increase in LVEF (WMD: 2.05; 95% CI: 1.64–2.46; *p* < 0.001) without significant heterogeneity (*I* ^*2*^ = 11.4%, *p* = 0.301) (Figure 6). Meta-regression analysis demonstrated that there was no significant positive correlation between the effect of GF on LVEF and baseline LVEF (regression = 0.81, *p* = 0.429) (Figure 7). And subgroup analyses showed that GF therapy increased LVEF both in baseline LVEF ≥50% (WMD 2.81; 95% CI: 2.21–3.42, *p* = 0.630) and baseline LVEF <50% (WMD 1.42; 95% CI: 0.88–1.97, *p* = 0.865). In addition, GF therapy increased LVEF only during short-time follow-up (<1 year) (WMD: 2.05; 95% CI: 1.64–2.46; *p* < 0.001) (Supplementary Figures S13–S17; Table 2).

#### 3.5.3 Canadian cardiovascular society angina class

**FIGURE 8:**
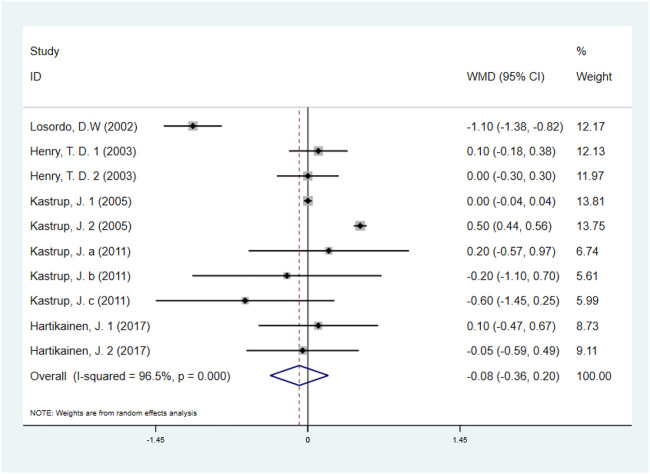
Forest plot for CCS angina class, GF vs. control. CCS, Canadian Cardiovascular Society; RR, relative risk; CI, confidence interval; ID, identification.

**FIGURE 9:**
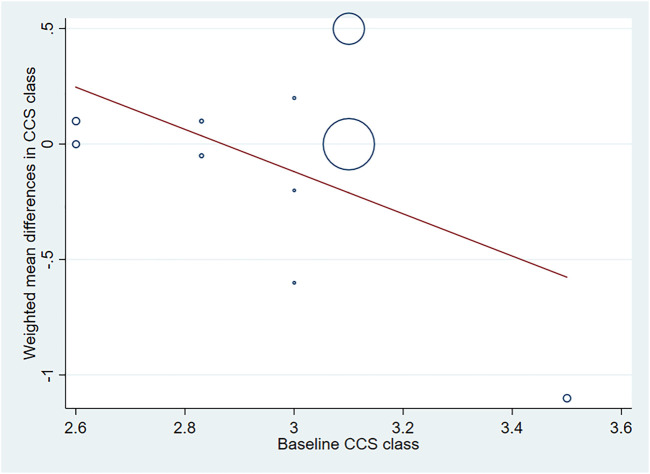
Meta-regression plots of the weighted mean difference in CCS angina class according to baseline CCS class (*p* = 0.127). CCS, Canadian Cardiovascular Society.

Five studies with 531 IHD patients (Losordo et al., 2002; Henry et al., 2003; Kastrup et al., 2005; Kastrup et al., 2011; Hartikainen et al., 2017) (291 individuals in GF group vs. 240 individuals in placebo group), evaluated the effect of GF on CCS angina class. The results demonstrated that there was no significant difference on CCS angina class between GF group and control group (WMD: −0.08, 95% CI: −0.36 to 0.20, *p* = 0.560) (Figure 8). Obvious heterogeneity between studies was observed (*I* ^*2*^ = 96.5%, *p* < 0.001). Sensitivity analyses showed no obvious changes in overall effect size with the summary WMDs ranged from −0.175 (95% CI: −0.481 to 0.131) to 0.069 (95% CI: −0.195–0.332), when individual studies were eliminated from the analysis. Subgroup analyses were carried out based on type of IHD, injection methods, follow-up duration and baseline CCS angina class, and the results indicated that those factors did not influence the final effect estimates (Supplementary Figures S18–S21; Table 2). Meta-regression analysis showed no statistical correlation between baseline CCS angina class and WMD in CCS angina class (regression = −1.70, *p* = 0.127) (Figure 9).

#### 3.5.4 Publication bias

**FIGURE 10:**
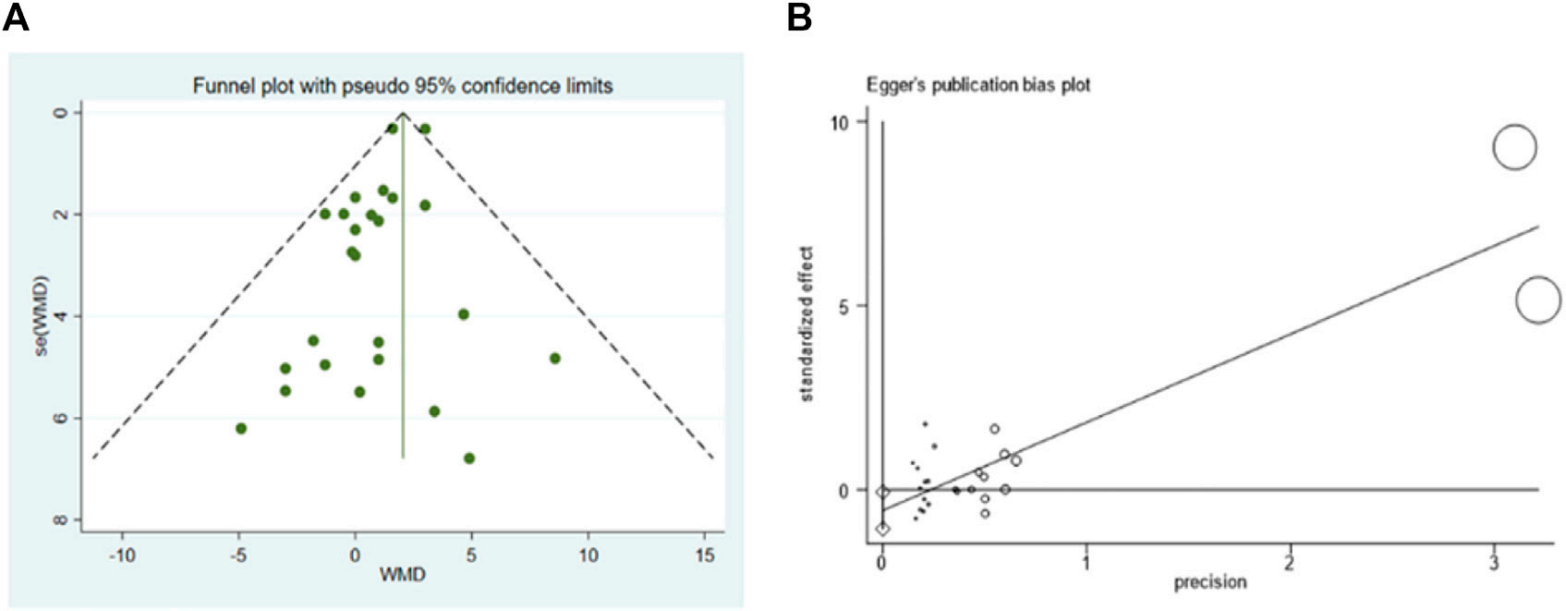
Publication bias. **(A)**, funnel plot; **(B)**, egger test (*p* > 0.05).

According to the visual inspection of funnel plot, a slight asymmetry was observed in the analysis for the effects of GF on LVEF. This was further confirmed by a significant Egger’s test (*p* < 0.001) (Figure 10). The application of the trim-and-fill method did not change the effect size (WMD 2.051; 95% CI: 1.645–2.457, *p* < 0.001) (Supplementary Figures S22). The funnel plots created for the visual analysis of the publication bias are presented in Figure 10.

## 4 Discussion

To our knowledge, the present meta-analysis is the first time to evaluate the effects of GF for therapeutic angiogenesis on IHD patients. The results showed that GF for therapeutic angiogenesis improved LVEF detected by echocardiography, rather than decreased all-cause mortality, MACE and revascularization during follow-up period. Furthermore, GF also did not improve the CCS angina class. Subgroup analysis showed that GF for therapeutic angiogenesis decreased LVEF only in short-term follow-up (<1 year) independent of baseline LVEF. Overall, these evidences supported that GF for therapeutic angiogenesis might be beneficial in improving cardiac function in short-term follow-up, however, they are not effective in decreasing hard endpoints, such as all-cause mortality and MACE.

Developing extensive collateral circulation in ischemic myocardium is a promising therapy for treating IHD. Even though antiplatelet agent and statin are cornerstones for treating IHD, however, previous clinical studies showed that statin and aspirin was effective in decreasing VEGF levels and have no effects on promoting angiogenesis (Dworacka et al., 2014; Cheng et al., 2015). Recently, animal studies showed that VEFG for therapeutic angiogenesis could promotes collateral circulation in mouse heart by recruiting endothelial progenitor cells, and subsequently rescue myocardial tissue after an ischemic insult (Mallick et al., 2022). Transforming growth factor beta (TGF-β1) induces pro-reparative phenotypic changes in epicardial cells in mice after myocardial infarction (Dergilev et al., 2021). However, in the present meta-analysis, we collect most comprehensive data regarding the effects of GF on all-cause mortality, MACE and revascularization, and we found that GF did not decrease the rate of all-cause mortality, MACE and revascularization. Low heterogeneity was observed regarding these outcomes, which increased the robustness of the results. For finding specific population might be beneficial from GF therapy, the subgroup analysis was performed. And we found that the results were stable independent of IHD, categories of growth factors, injection methods and follow-up duration. The effects of GF on CCS angina class were also evaluated, and we found GF therapy could not decrease CCS angina class. Taking together, the results of these studies supported that GF therapy may not effective in improving the prognosis of IHD.

LVEF is a quantitative marker to evaluate cardiac systolic function. previous study showed that patients with preserved left ventricular ejection fraction had lower one and 3-year mortality rates as compared with reduced left ventricular ejection fraction regardless of the acute coronary syndrome period onset (Yahud et al., 2020). Our results showed that GF for therapeutic angiogenesis could increase LVEF by 2.05%. And the subgroup analysis found that GF therapy was only effective during a short-term follow-up (<1 year). The metabolism of GF might lead to a transient effect on cardiac function. What’s more, angiogenesis is a complex biological process that involves degradation of the vascular basement membrane, endothelial cell proliferation, vascular sprouting, lumen formation, and stabilization and maturation of the vascular network. Relying only on a single injection of a singular GF may promote angiogenesis in a short period of time (<1 year), thus improving cardiac function and increasing LVEF, however, the long-term (≥1 year) efficacy on cardiac function is not significant. Moreover, GF showed notable improvement in cardiac function in SCHD and MIHF with stable disease, while it had no efficacy in refractory CAD or STEMI with critical disease. For specific GF categories, VEGF and HGF showed dramatic improvement in LVEF, while EPO and G-CSF had no obvious efficacy. Interestingly, HGF has a beneficial synergistic effect with VEGF. There is a study identified that HGF prominently promotes the effects of VEGF on angiogenesis *via* the ets-1 pathway (Tomita et al., 2003). Furthermore, in respect to the injection methods, gene transfer therapy is superior to protein injection therapy, because gene transfer can increase LVEF by 2.95%, transcutaneous endocardium injection of GF protein only increased by 1.60%, while intravenous injection, myocardial injection and subcutaneous injection of GF protein could not increase LVEF. We consider that GF should be used more often in the treatment of IHD patients with stable disease conditions. In terms of treatment methods, a combination of multiple synergistic GFs application and more frequent administration can be used in future clinical practice, and the gene transfer mode of delivery is more effective.

There are some limitations in our study. First, there are different injection method of GF, including intramyocardial injections and intracoronary infusion, which might lead to clinical heterogeneity. Thus, we performed subgroup analyses based on injection method, and the effect sizes did not change. Second, the sample size of included studies is relatively small and might lead to less robust results, we would update the meta-analysis when large-scale clinical studies publish. Third, the long-term persistence of the treatment effects is unknown. Most of the trials ranged in duration from 3 to 12 months. Fourth, obvious publication bias was found from egger’s test, which might influence the credibility of present results. Thus, trim-and-fill method was used to evaluate the corrected effect size, and we found that the effect size remained unchanged.

## 5 Conclusion

Even though GF for therapeutic angiogenesis was beneficial for increasing LVEF during short-term follow-up (<1 year), the therapy was not efficacious in decreasing all-cause mortality and MACE in IHD patients.
