# Supplementary material for: Growth factor for therapeutic angiogenesis in ischemic heart disease: A meta-analysis of randomized controlled trials
Source: Front Cell Dev Biol. 2022 Dec 9;10:1095623. doi: 10.3389/fcell.2022.1095623 (PMC9780500; doi:10.3389/fcell.2022.1095623)
Supplement: Supplementary file 1 [file DataSheet1.PDF]

Growth factor for therapeutic angiogenesis in ischemic heart disease a meta-analysis of randomized controlled study

Ling Tan[1†],Lin-zi Long[1†],Hong-zheng Li[1,3†], Wen-wen Yang[1], Yu-xuan Peng[1,3], Jie-ming Lu[1,3], Fei-fei Liao[1,3], Xiao-chang Ma[1,2], Hua Qu[1,2]*, Chang-geng Fu[1,2]* Shan-shan Zhang[4]*

[1]Xiyuan Hospital, China Academy of Chinese Medical Sciences
[2]National Cardiovascular Clinical Medical Research Center of TCM

[3] Graduate School of Beijing University of Chinese Medicine Beijing Xibeiwang

[4]Town Community Health Service Center, China

*** Correspondence:**
Shan-shan Zhang: zhangshanshan0533@163.com; Chang-geng Fu: fucgbs@163.com;
Hua Qu:hua_qu@yeah.net

[†]These authors contributed equally to this work and share first authorship.

Table S1. Search strategy

#	Query
**A: Search strategy in MEDLINE**	
#	Query
1#	Vascular endothelial growth factor[Title/Abstract] OR VEGF [Title/Abstract] OR Placental growth factor [Title/Abstract] OR PLGF [Title/Abstract] OR Fibroblast growth factor [Title/Abstract] OR FGF [Title/Abstract] OR Hepatocyte growth factor [Title/Abstract] OR HGF [Title/Abstract] OR Platelet-derived growth factor[Title/Abstract] OR PDGF [Title/Abstract] OR Angiopoietin [Title/Abstract] OR Ang[Title/Abstract] OR Erythropoietin [Title/Abstract] OR EPO [Title/Abstract]
2#	ischemic heart disease [Title/Abstract] OR coronary artery disease [Title/Abstract] OR coronary heart disease [Title/Abstract]
3#	randomized controlled trial[Title/Abstract] OR clinical trial[Title/Abstract]
4#	1# and 2# and 3#
**B: Search strategy in Embase**	
#	Query
1#	'Vascular endothelial growth factor':ab,ti OR 'VEGF': ab,ti OR 'Placental growth factor':ab,ti OR 'PLGF': ab,ti OR 'Fibroblast growth factor': ab,ti OR 'FGF': ab,ti OR 'Hepatocyte growth factor': ab,ti OR 'HGF' :ab,ti OR 'Platelet-derived growth factor': ab,ti OR 'PDGF':ab,ti OR 'Angiopoietin': ab,ti OR 'Ang': ab,ti OR 'Erythropoietin':ab,ti OR 'EPO':ab,ti
2#	'ischemic heart disease':ab,ti OR 'coronary artery disease':ab,ti OR 'coronary heart disease':ab,ti
3#	'randomized controlled trial':ab,ti OR 'clinical trial':ab,ti
4#	1# and 2# and 3#
**C: Search strategy in CENTRAL**	
#	Query
1#	(vascular endothelial growth factor): ti,ab,kw OR (VEGF): ti,ab,kw OR (Placental growth factor): ti,ab,kw OR (PLGF): ti,ab,kw OR (Fibroblast growth factor) : ti,ab,kw OR (FGF): ti,ab,kw OR (Hepatocyte growth factor): ti,ab,kw OR (HGF): ti,ab,kw OR (Platelet-derived growth factor): ti,ab,kw OR (PDGF) : ti,ab,kw OR (Angiopoietin): ti,ab,kw OR (Ang) : ti,ab,kw OR (Erythropoietin): ti,ab,kw OR (EPO) : ti,ab,kw
2#	(ischemic heart disease): ti,ab,kw OR ([Myocardial Ischemia): Mesh OR (coronary artery disease): ti,ab,kw OR (Coronary Artery Disease): Mesh OR (coronary heart disease): ti,ab,kw OR (Coronary Disease): Mesh
3#	(randomized-controlled trial): ti,ab,kw OR (clinical trial): ti,ab,kw
4#	1# and 2# and 3#

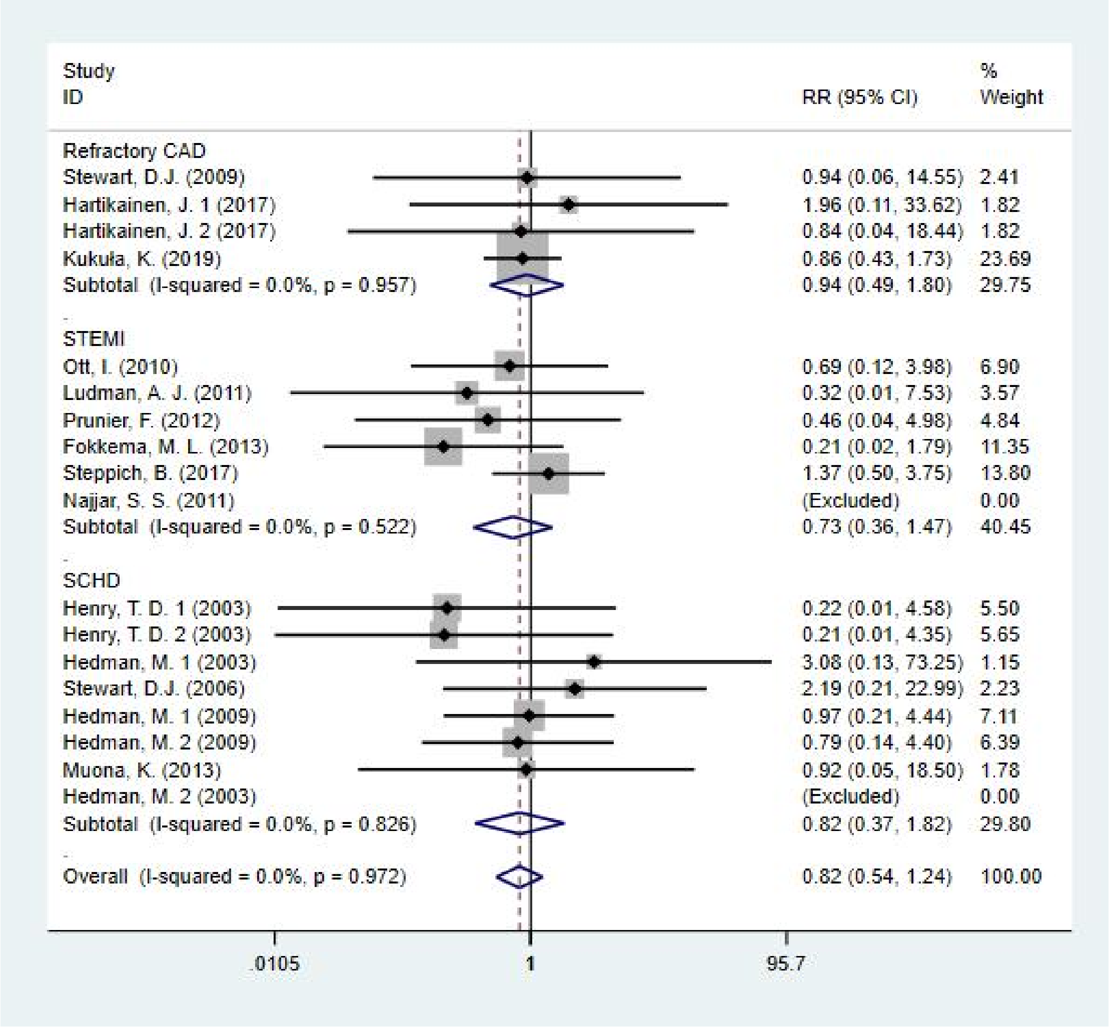

FIGURE S1 | Subgroup analysis of the effect of GF on All-cause mortality based on type of IHD. IHD: ischemic heart disease; RR: relative risk; CI: confidence interval; ID: identification.

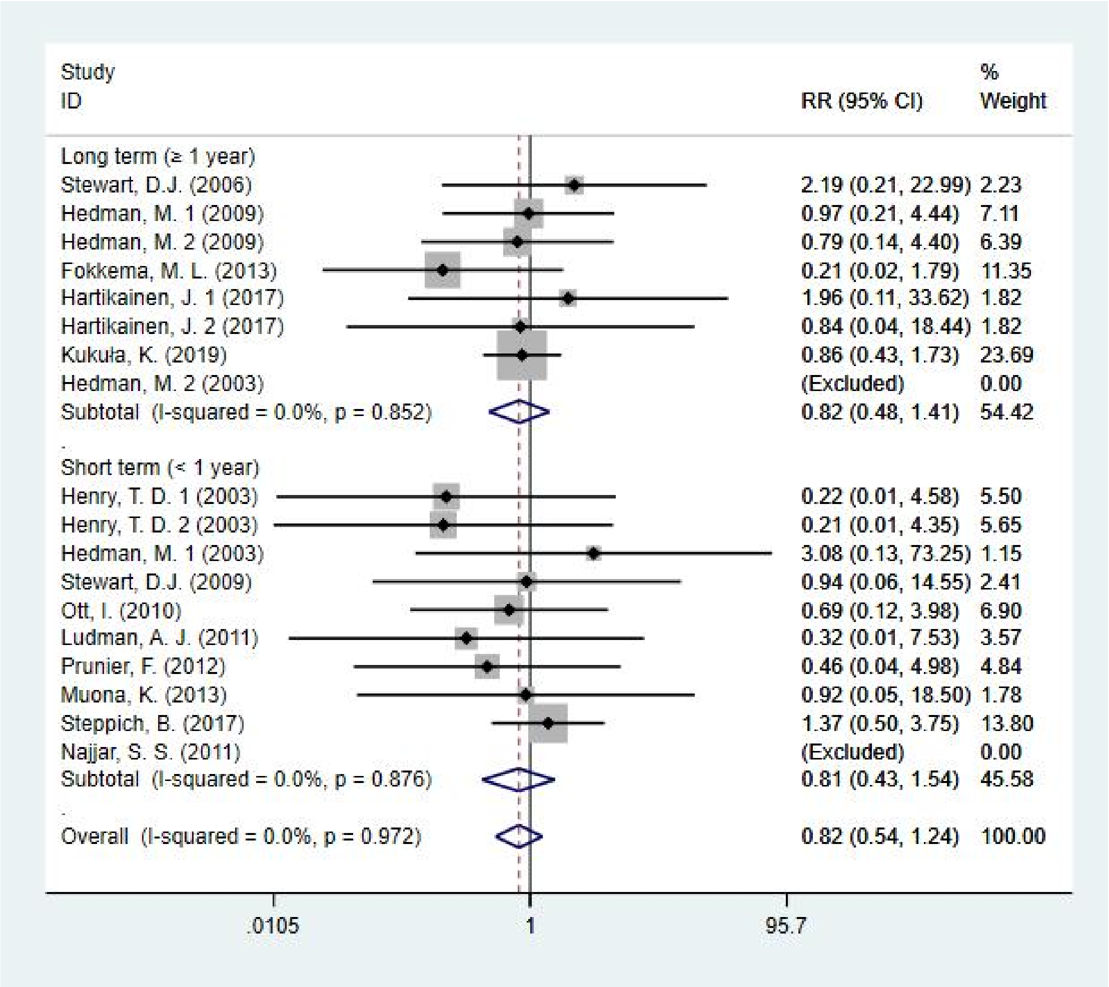

FIGURE S2 | Subgroup analysis of the effect of GF on All-cause mortality based on follow-up duration. RR: relative risk; CI: confidence interval; ID: identification.

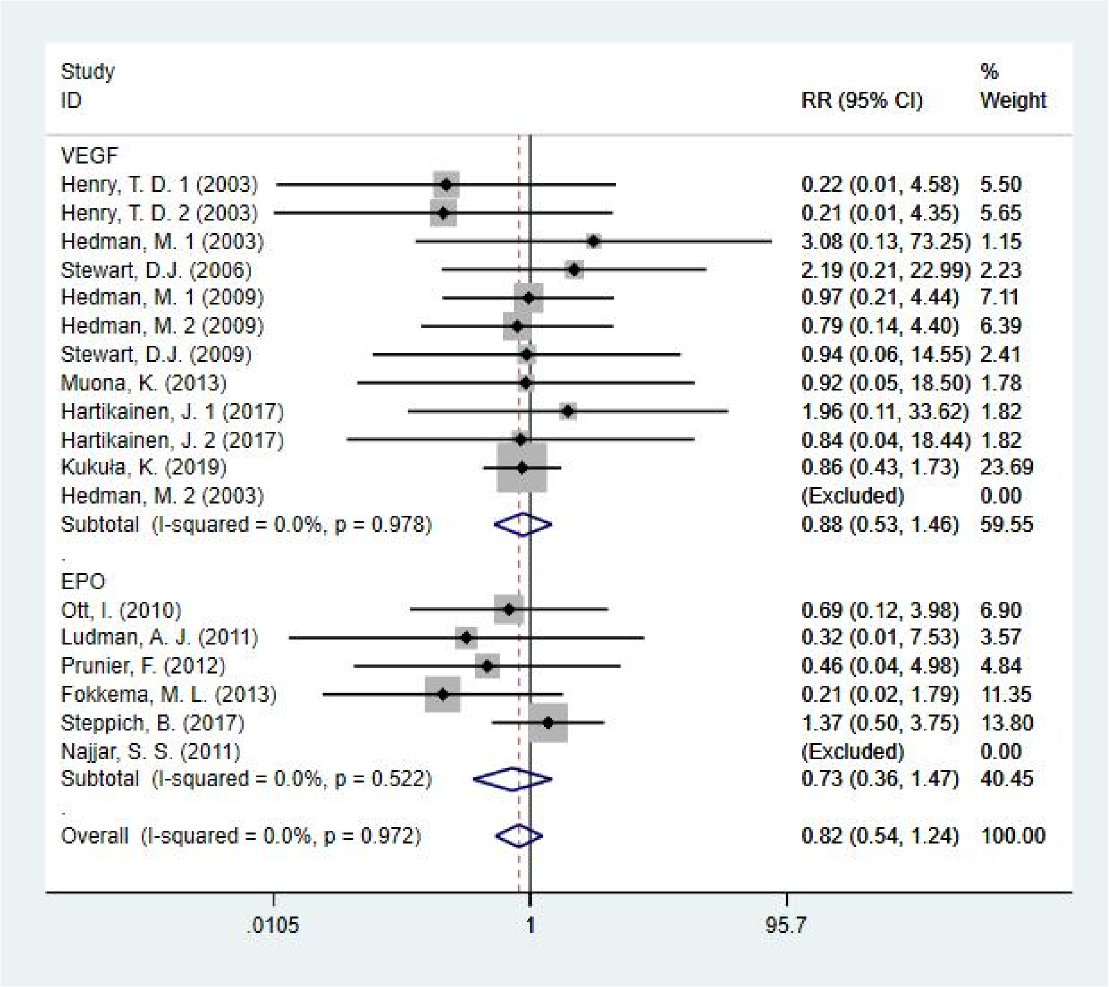

FIGURE S3 | Subgroup analysis of the effect of GF on All-cause mortality based on categories of growth factors. RR: relative risk; CI: confidence interval; ID: identification.

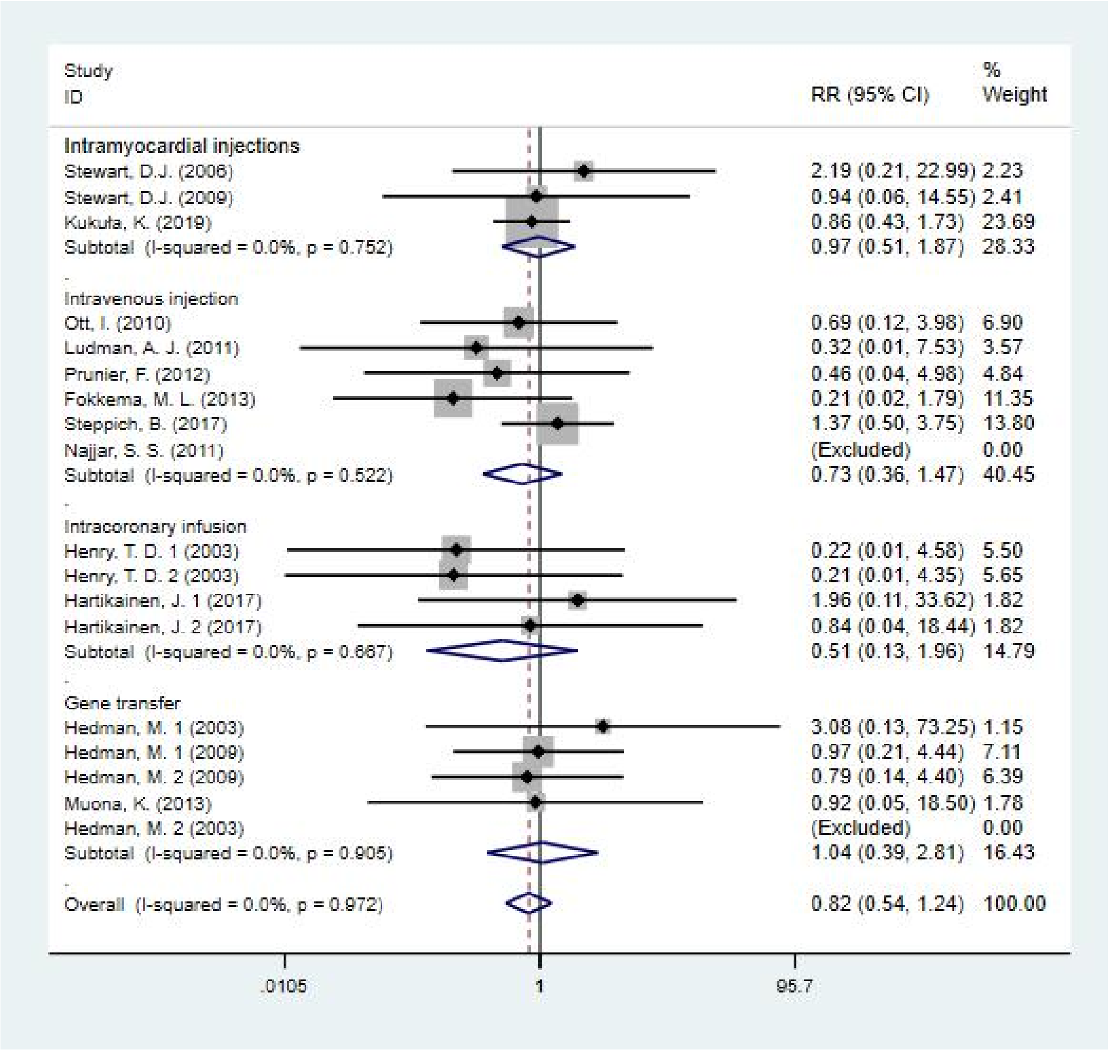

FIGURE S4 | Subgroup analysis of the effect of GF on All-cause mortality based on injection methods. RR: relative risk; CI: confidence interval; ID: identification.

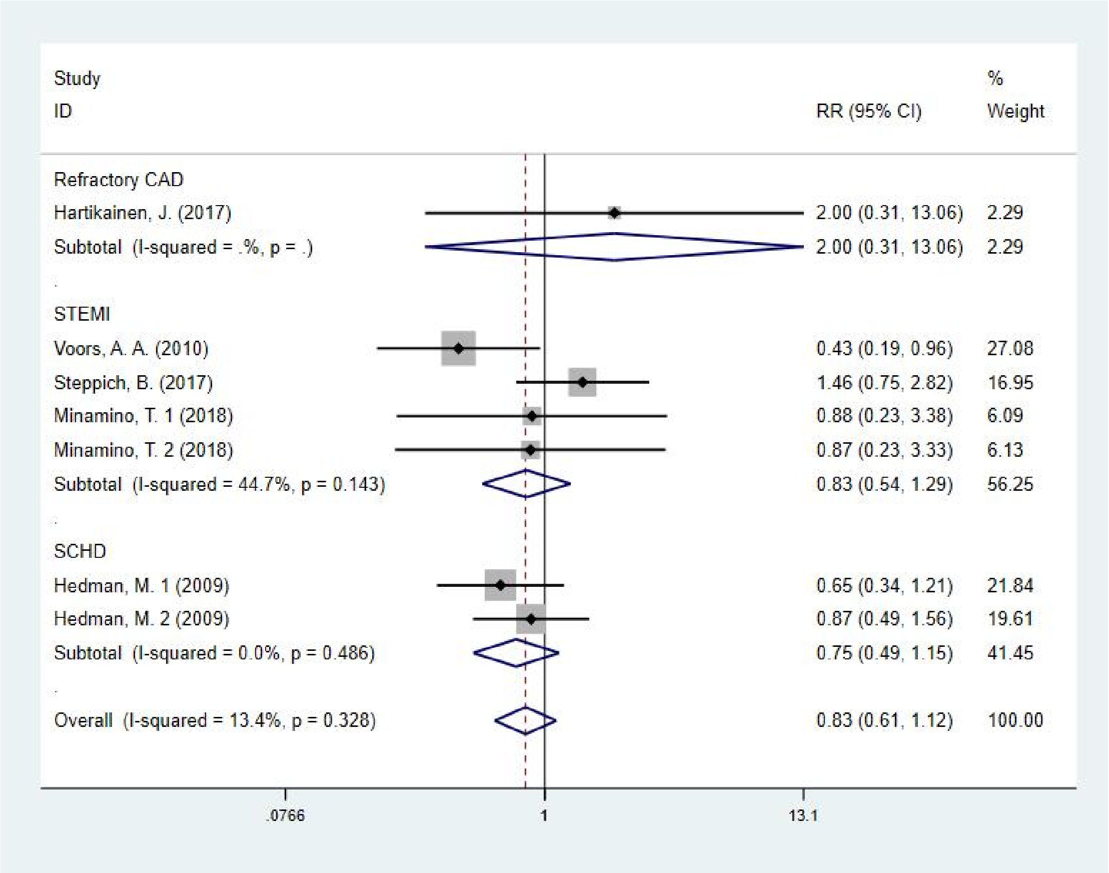

FIGURE S5 | Subgroup analysis of the effect of GF on MACE based on type of IHD. MACE: major adverse cardiovascular events; IHD: ischemic heart disease; RR: relative risk; CI: confidence interval; ID: identification.

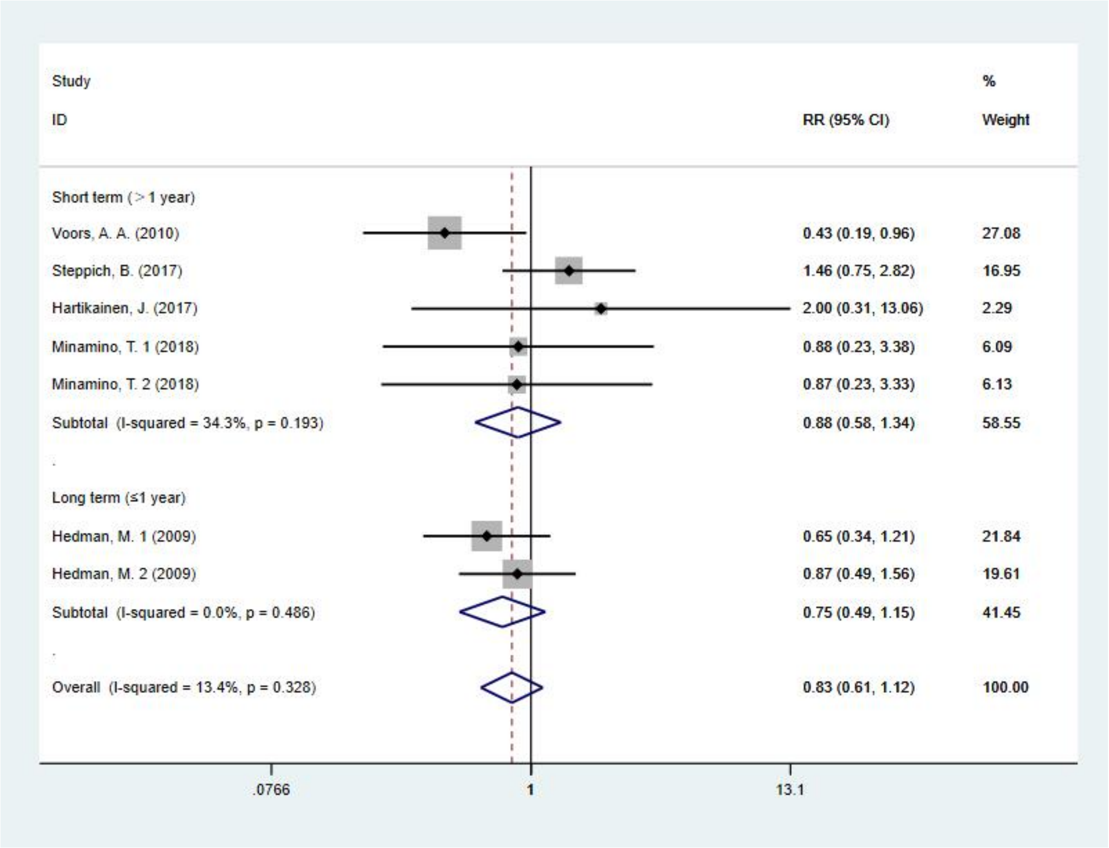

FIGURE S6 | Subgroup analysis of the effect of GF on MACE based on follow-up duration. MACE: major adverse cardiovascular events; RR: relative risk; CI: confidence interval; ID: identification.

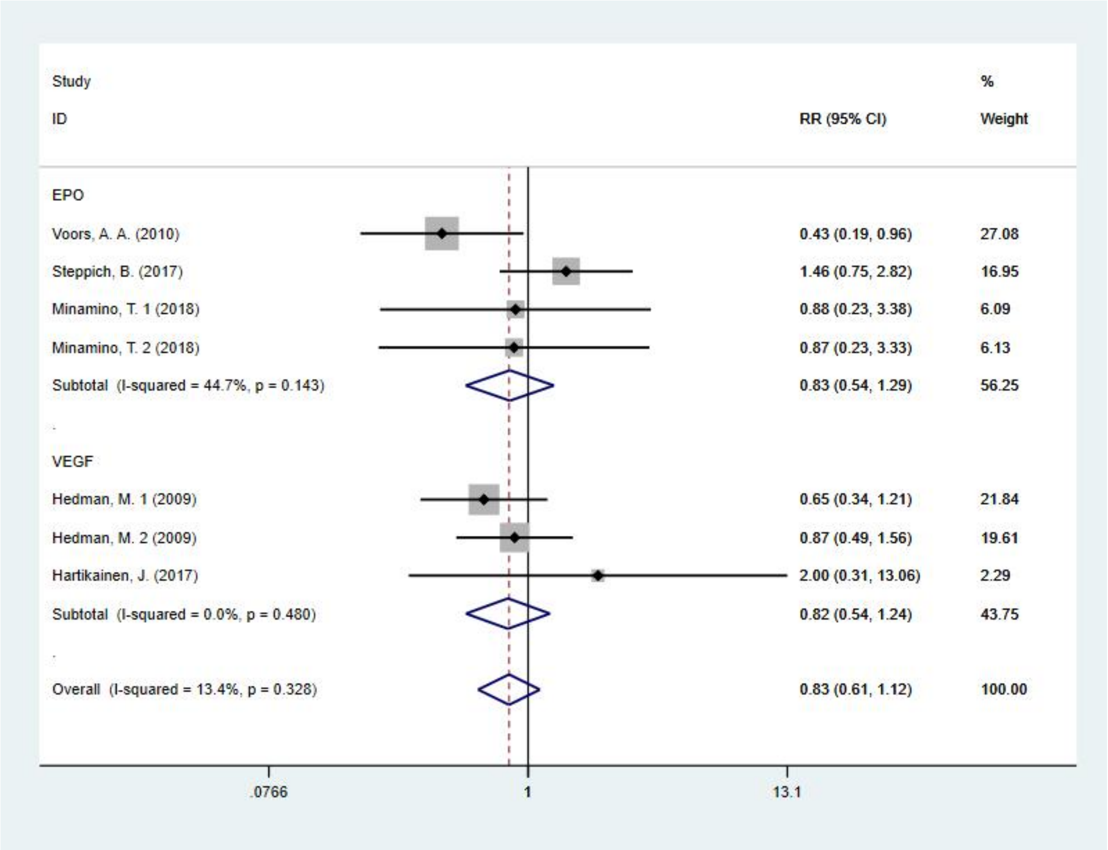

FIGURE S7 | Subgroup analysis of the effect of GF on MACE based on categories of growth factors. MACE: major adverse cardiovascular events; RR: relative risk; CI: confidence interval; ID: identification.

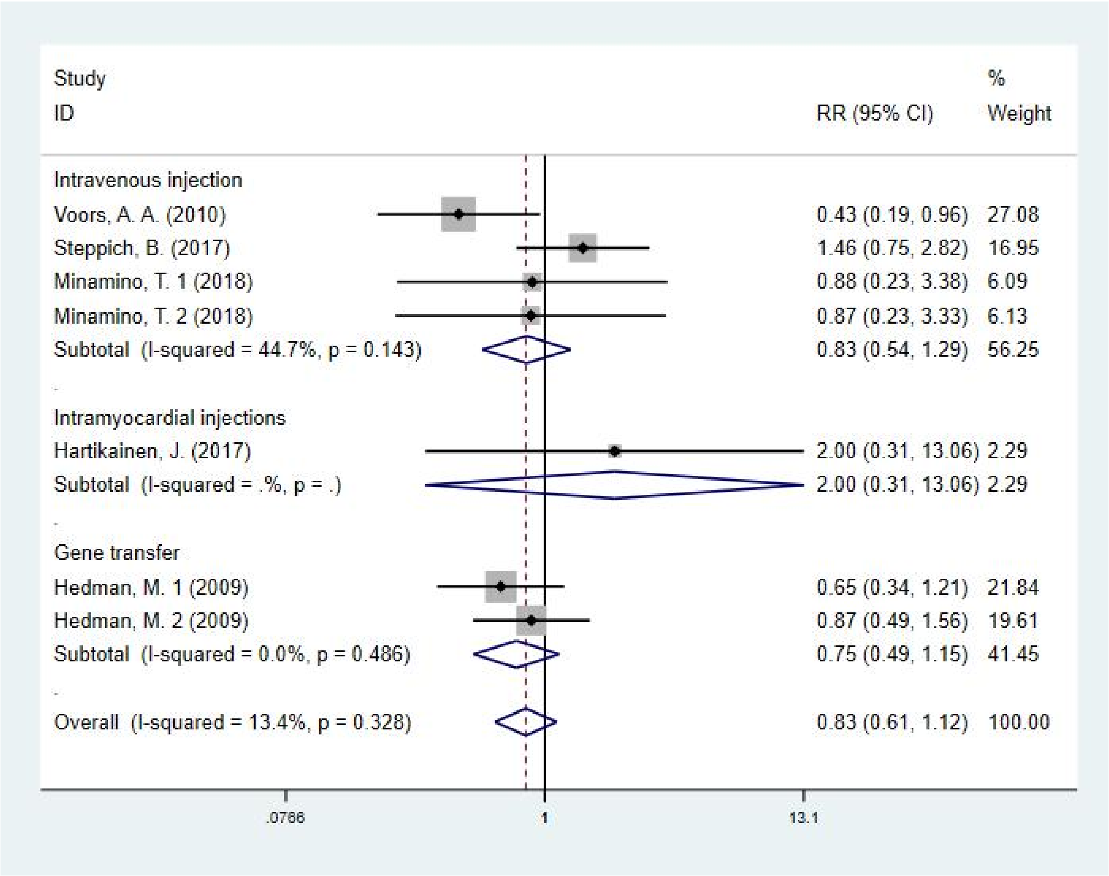

FIGURE S8 | Subgroup analysis of the effect of GF on MACE based on injection methods. MACE: major adverse cardiovascular events; RR: relative risk; CI: confidence interval; ID: identification.

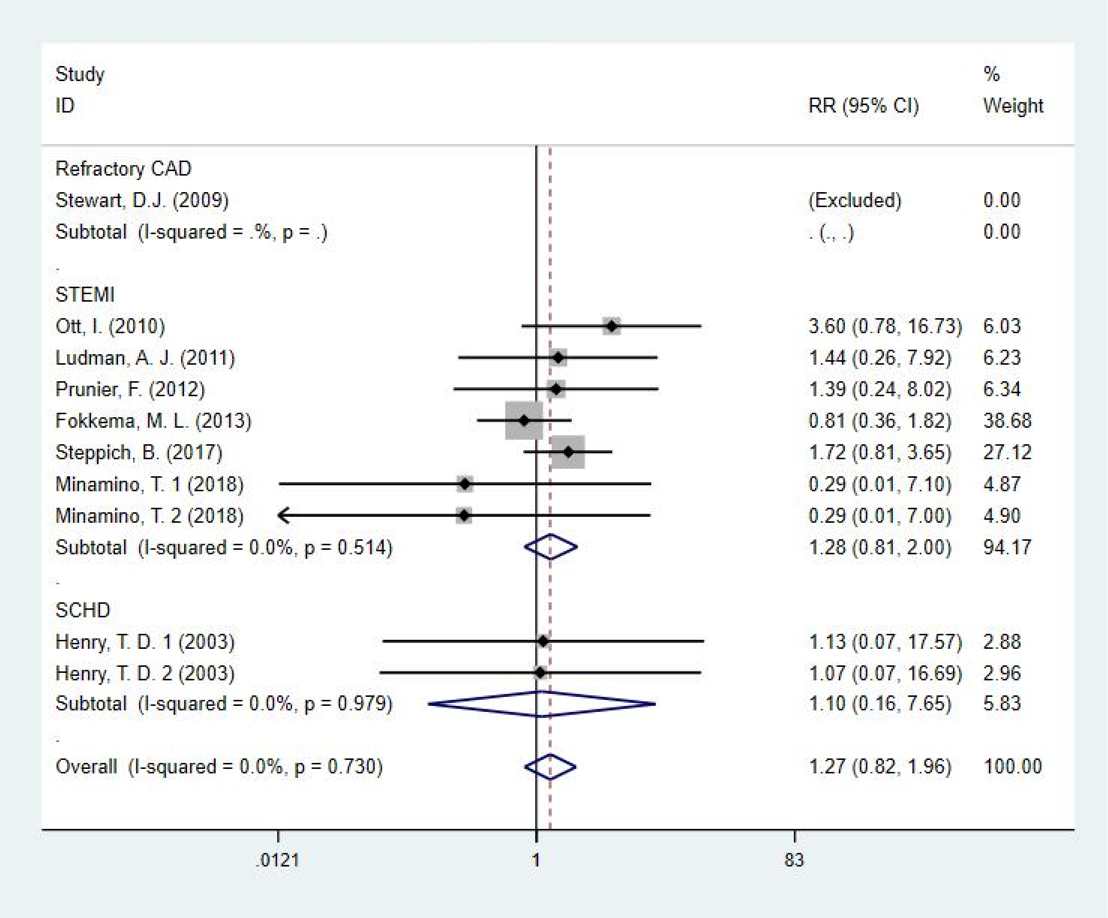

FIGURE S9 | Subgroup analysis of the effect of GF on revascularization based on type of IHD. IHD: ischemic heart disease; RR: relative risk; CI: confidence interval; ID: identification.

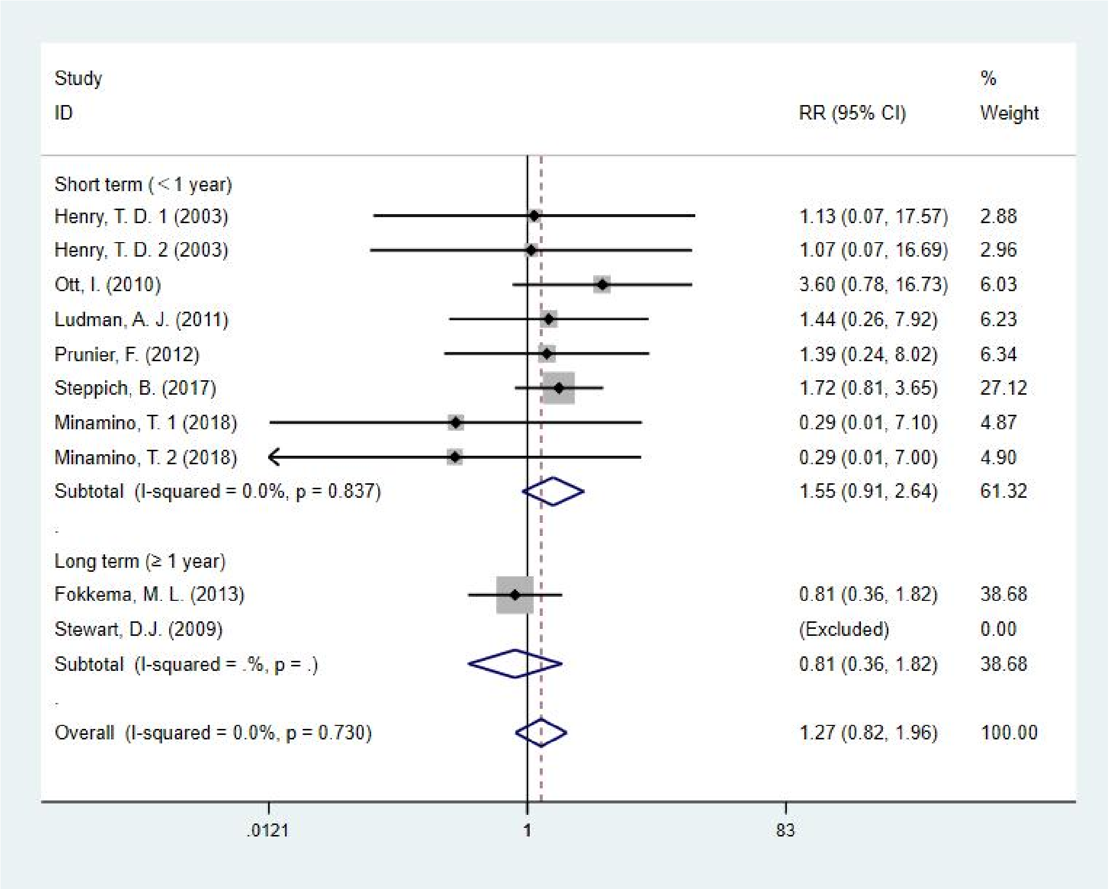

FIGURE S10 | Subgroup analysis of the effect of GF on revascularization based on follow-up duration. RR: relative risk; CI: confidence interval; ID: identification.

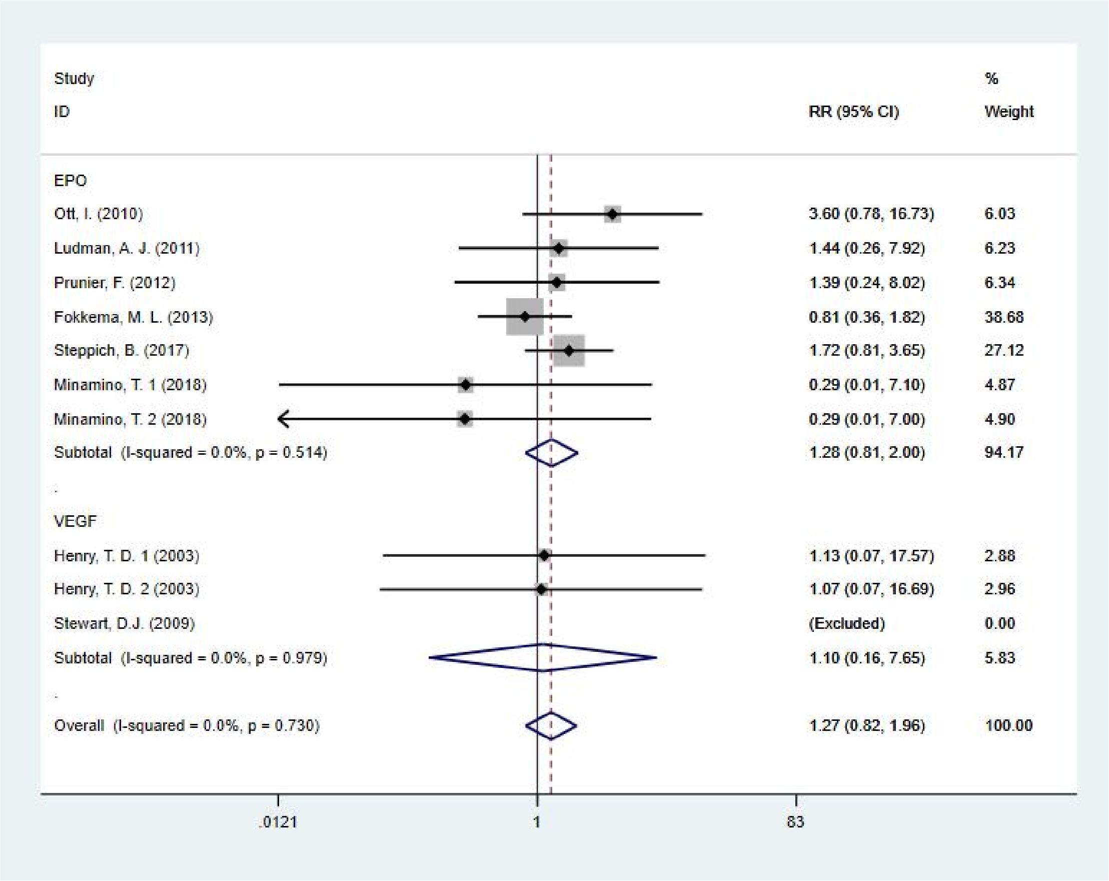

FIGURE S11 | Subgroup analysis of the effect of GF on revascularization based on categories of growth factors. RR: relative risk; CI: confidence interval; ID: identification.

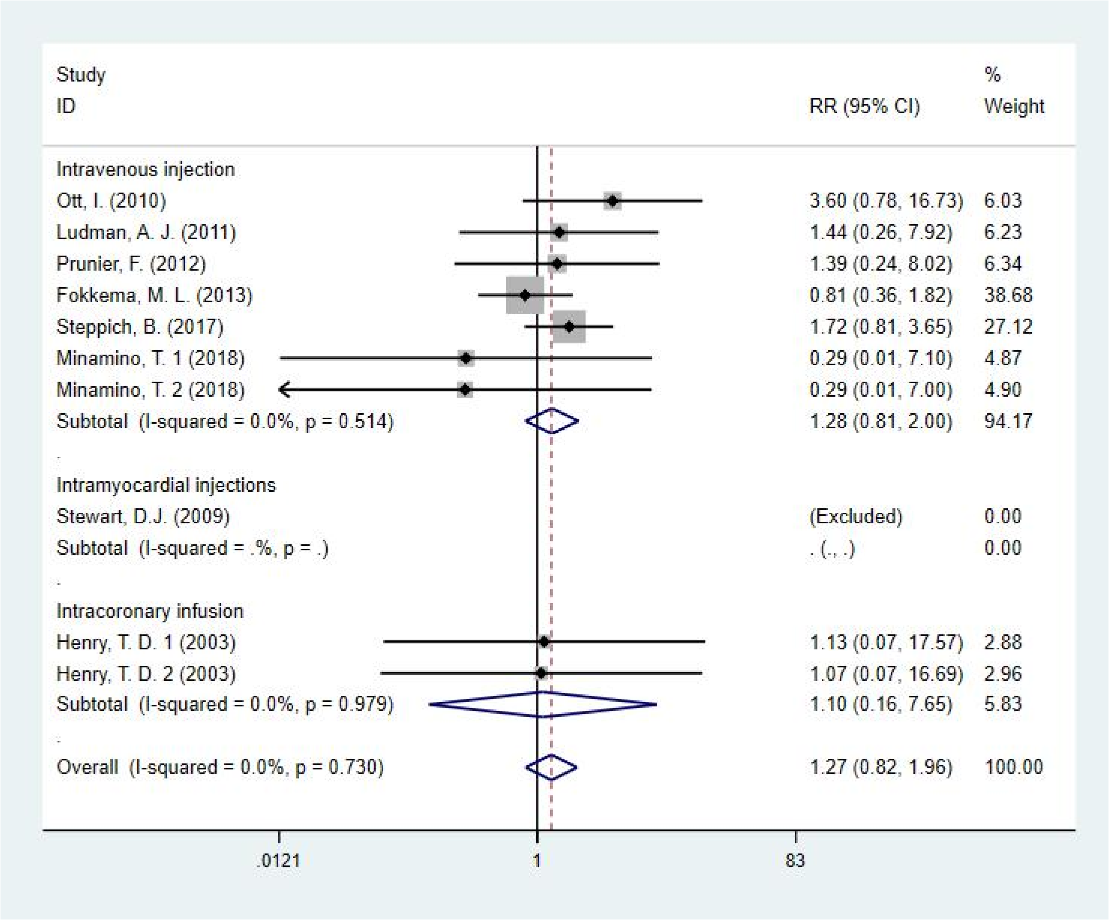

FIGURE S12 | Subgroup analysis of the effect of GF on revascularization based on injection methods. RR: relative risk; CI: confidence interval; ID: identification.

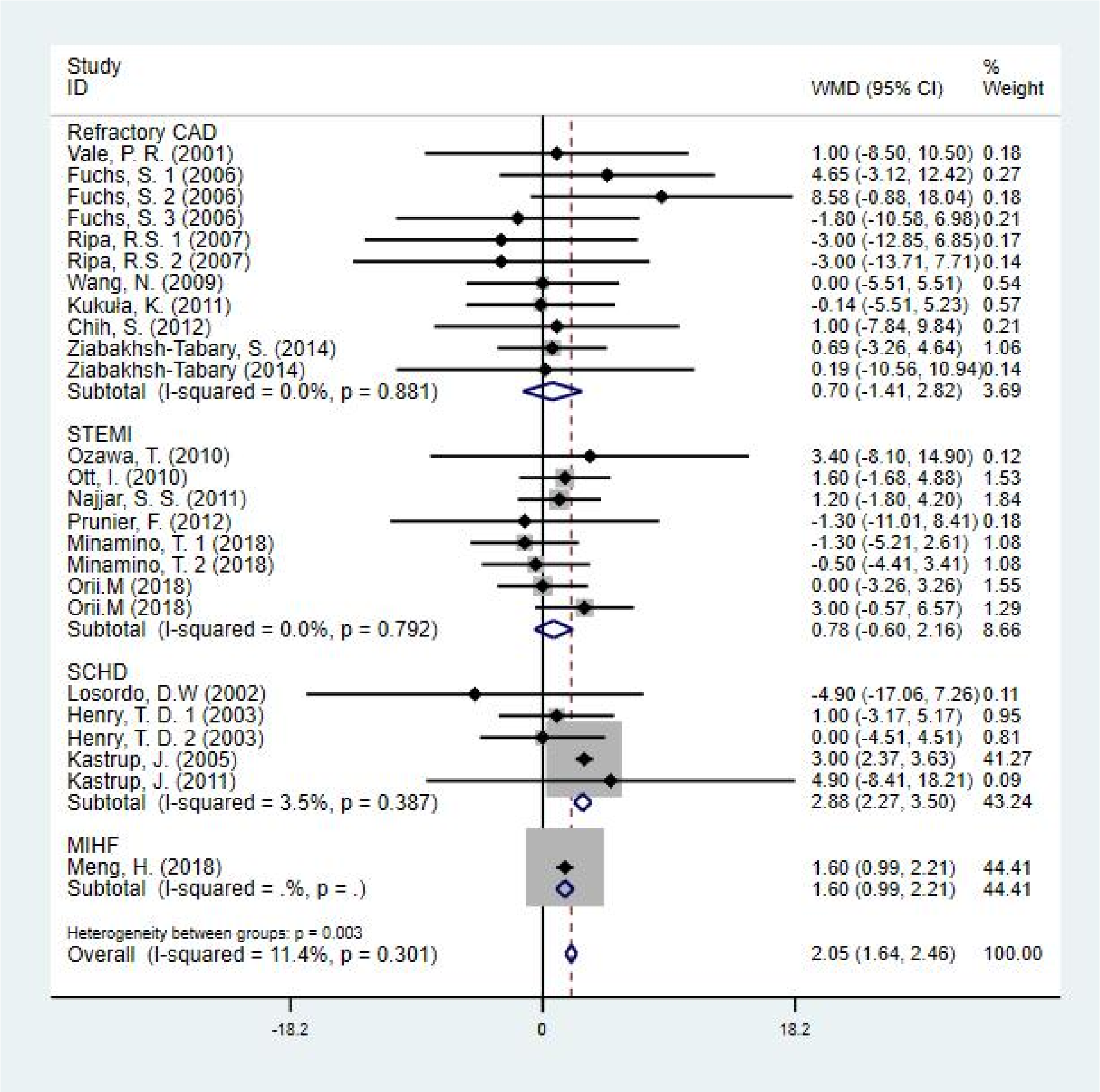

FIGURE S13 | Subgroup analysis of the effect of GF on LVEF based on type of IHD. LVEF: left ventricular ejection fraction; IHD: ischemic heart disease; WMD: weighted mean difference; CI: confidence interval; ID: identification.

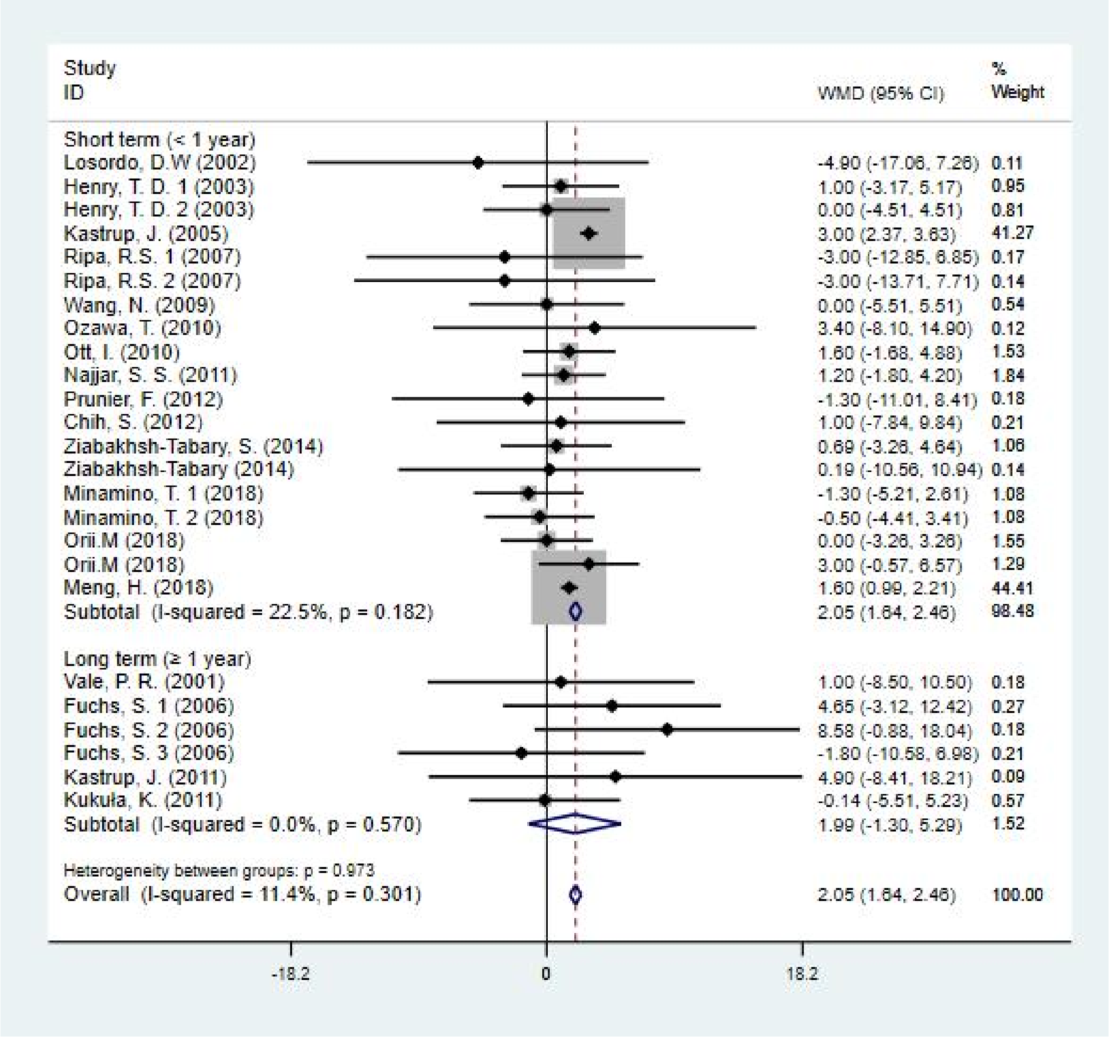

FIGURE S14 | Subgroup analysis of the effect of GF on LVEF based on follow-up duration. LVEF: left ventricular ejection fraction; WMD: weighted mean difference; CI: confidence interval; ID: identification.

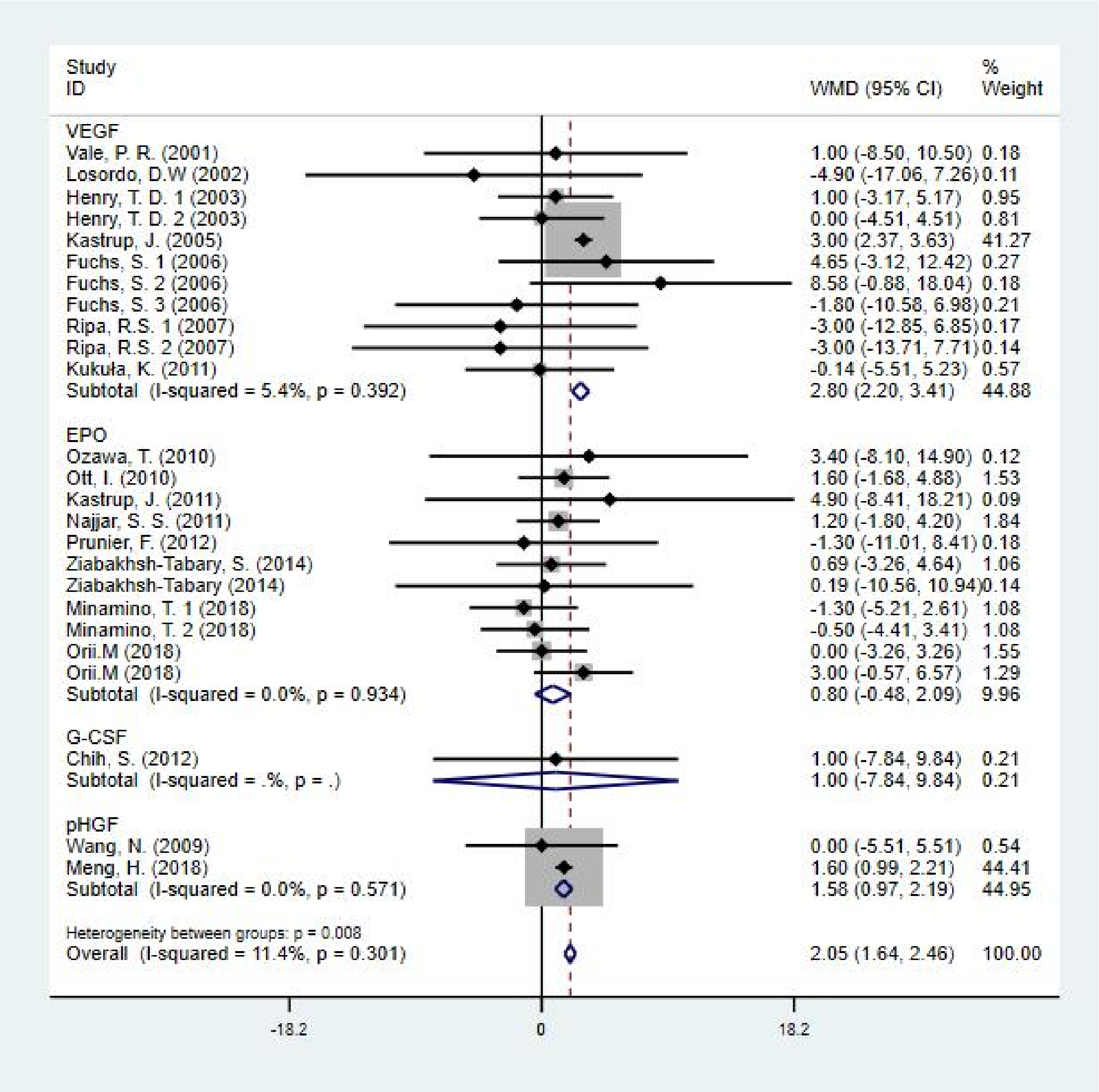

FIGURE S15 | Subgroup analysis of the effect of GF on LVEF based on categories of growth factors. LVEF: left ventricular ejection fraction; WMD: weighted mean difference; CI: confidence interval; ID: identification.

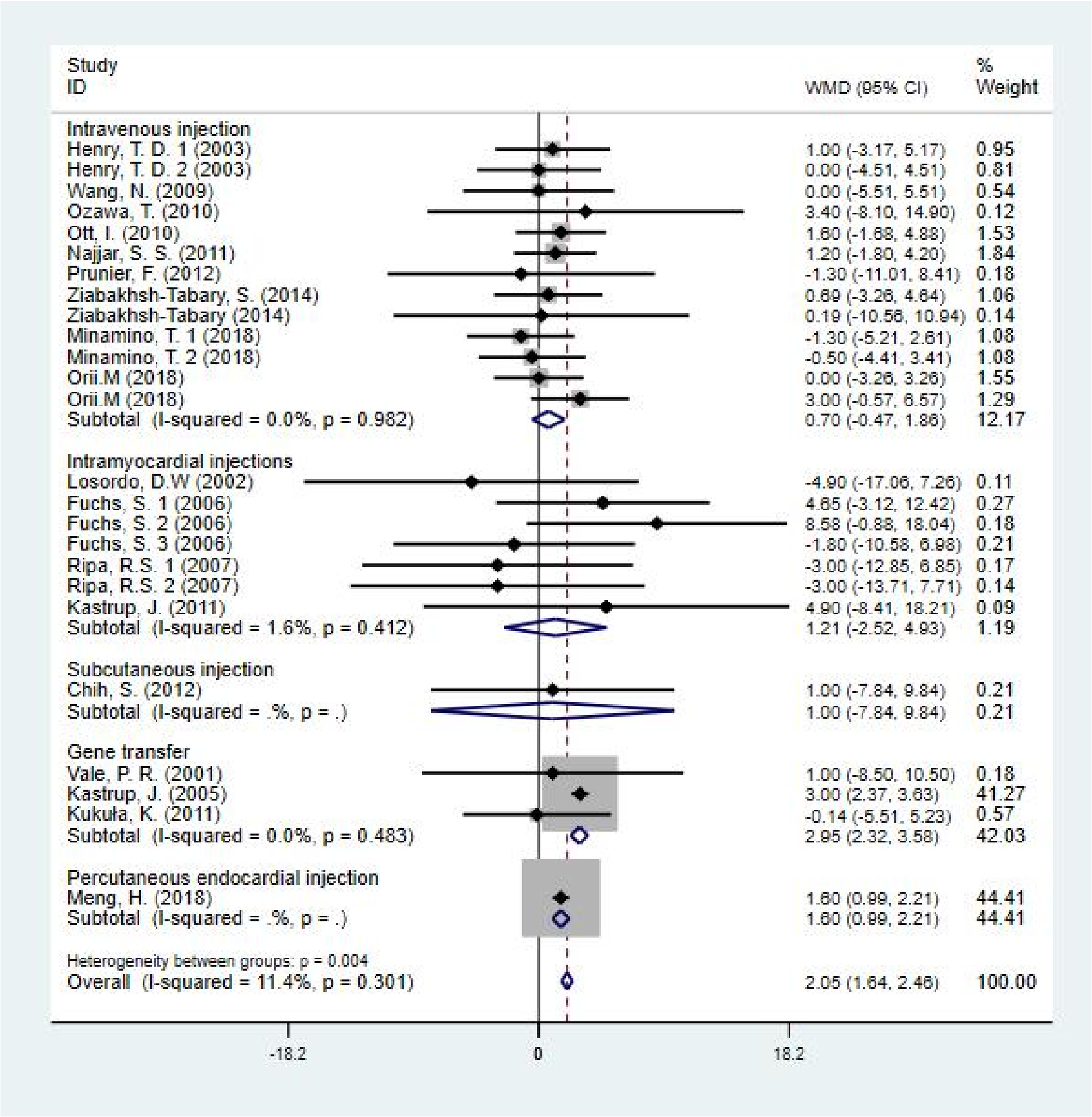

FIGURE S16 | Subgroup analysis of the effect of GF on LVEF based on injection methods. LVEF: left ventricular ejection fraction; WMD: weighted mean difference; CI: confidence interval; ID: identification.

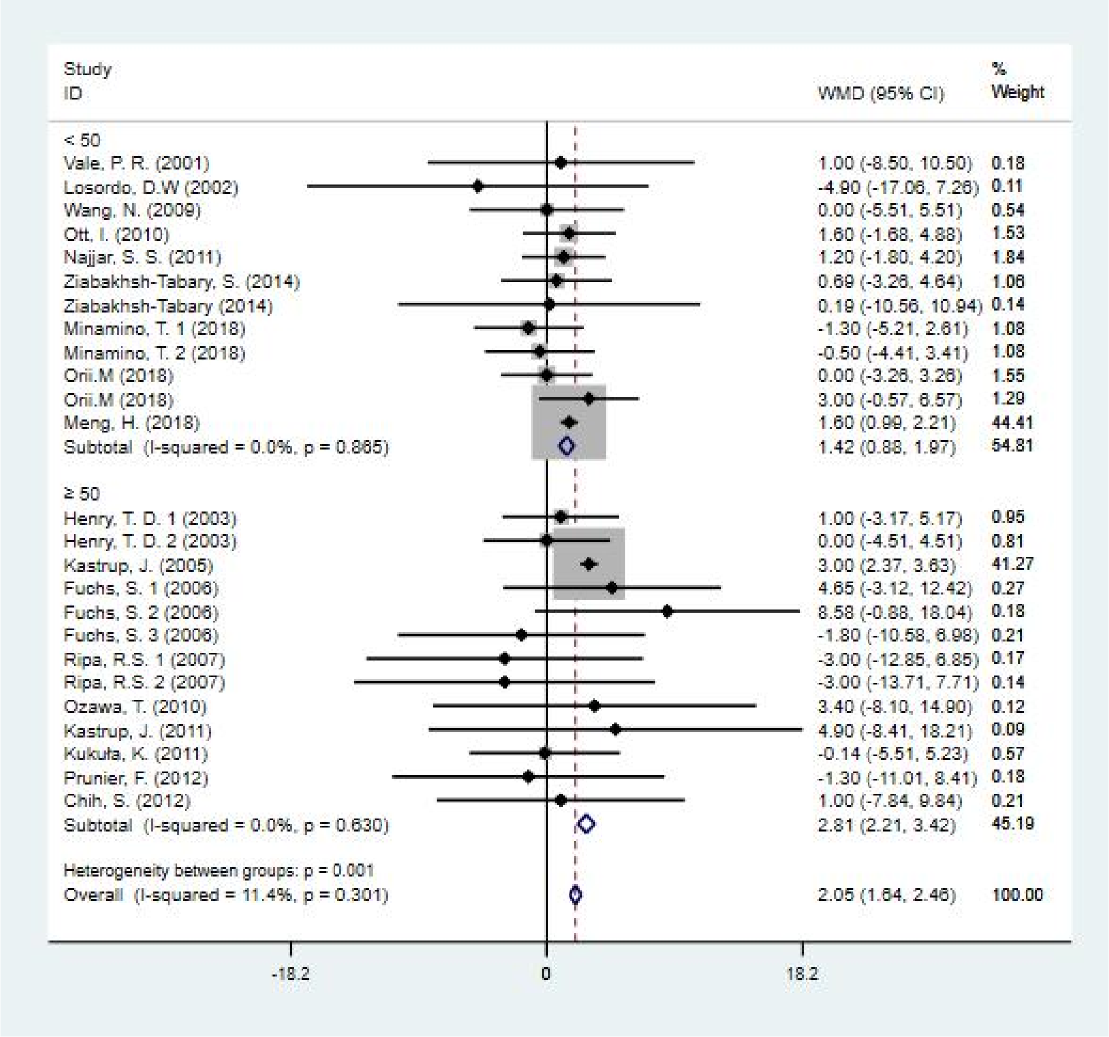

FIGURE S17 | Subgroup analysis of the effect of GF on LVEF based on Baseline LVEF values. LVEF: left ventricular ejection fraction; WMD: weighted mean difference; CI: confidence interval; ID: identification.

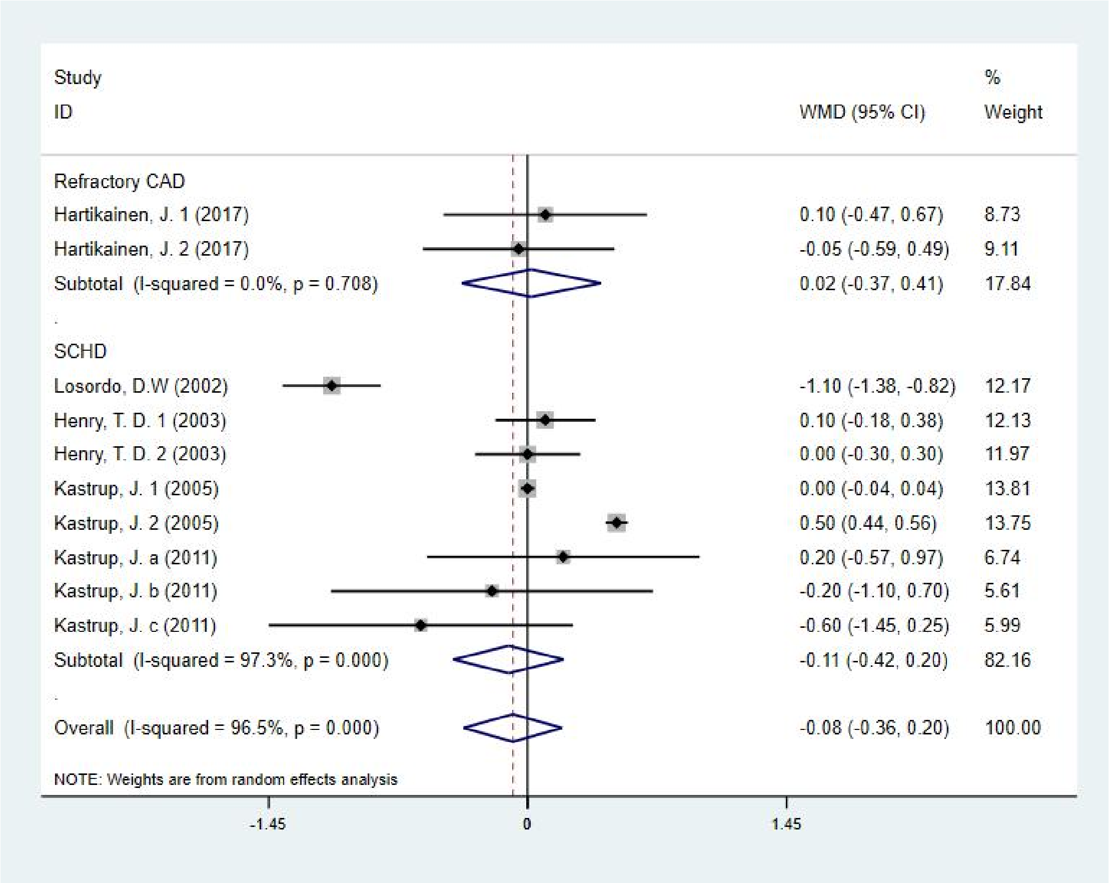

FIGURE S18 | Subgroup analysis of the effect of GF on CCS angina class based on type of IHD. CCS: Canadian Cardiovascular Society; IHD: ischemic heart disease; WMD: weighted mean difference; CI: confidence interval; ID: identification.

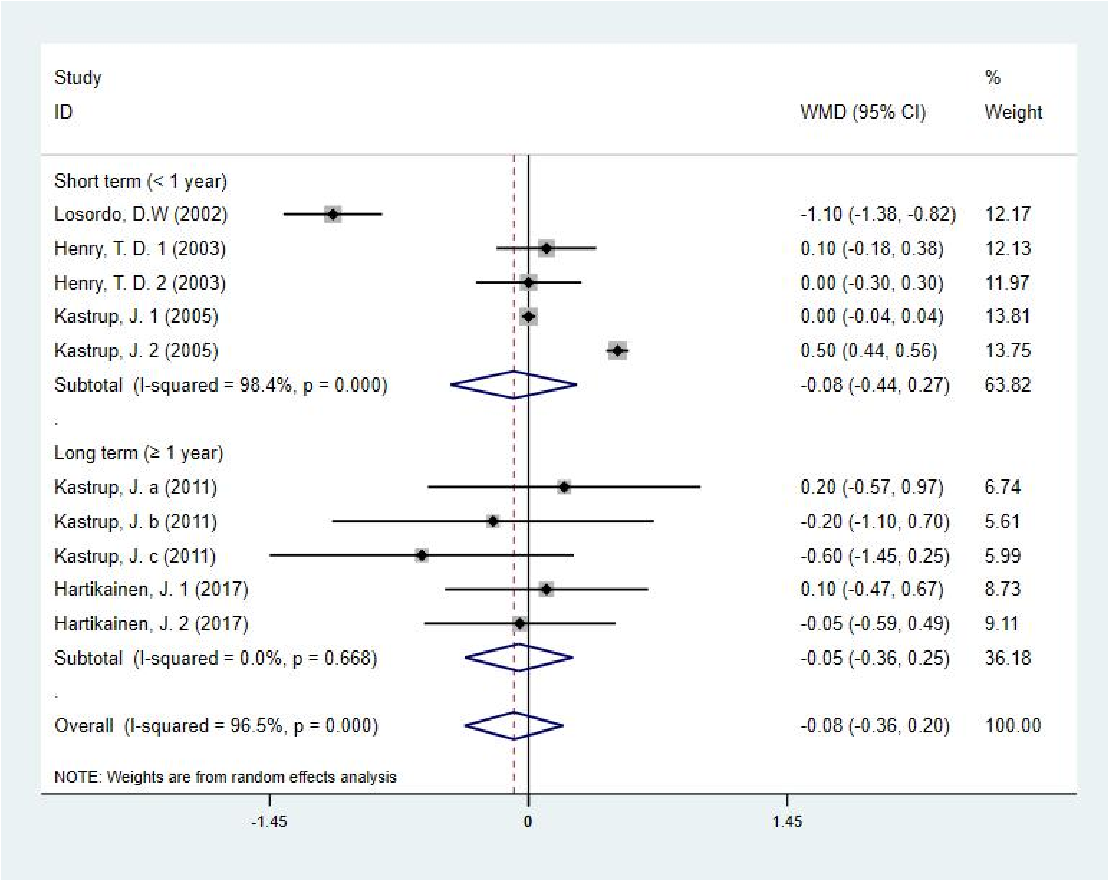

FIGURE S19 | Subgroup analysis of the effect of GF on CCS angina class based on follow-up time. CCS: Canadian Cardiovascular Society; WMD: weighted mean difference; CI: confidence interval; ID: identification.

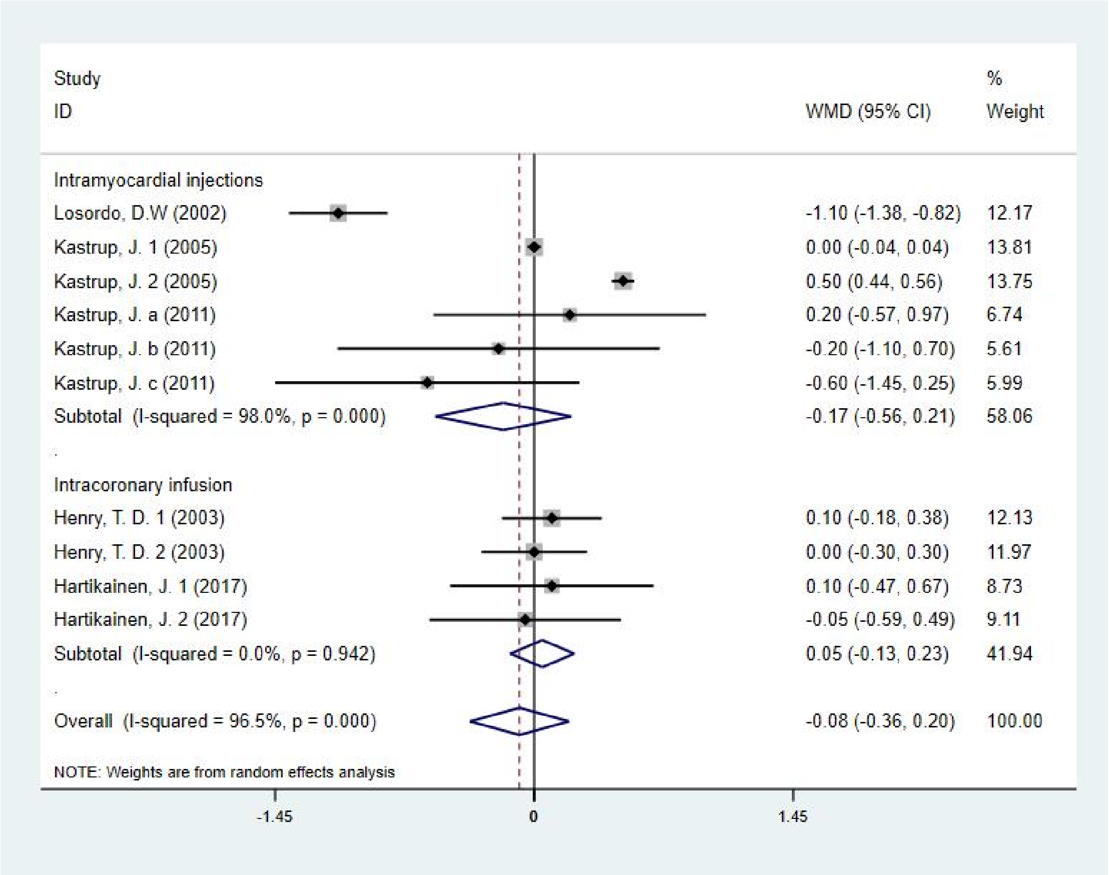

FIGURE S20 | Subgroup analysis of the effect of GF on CCS angina class based on injection methods. CCS: Canadian Cardiovascular Society; WMD: weighted mean difference; CI: confidence interval; ID: identification.

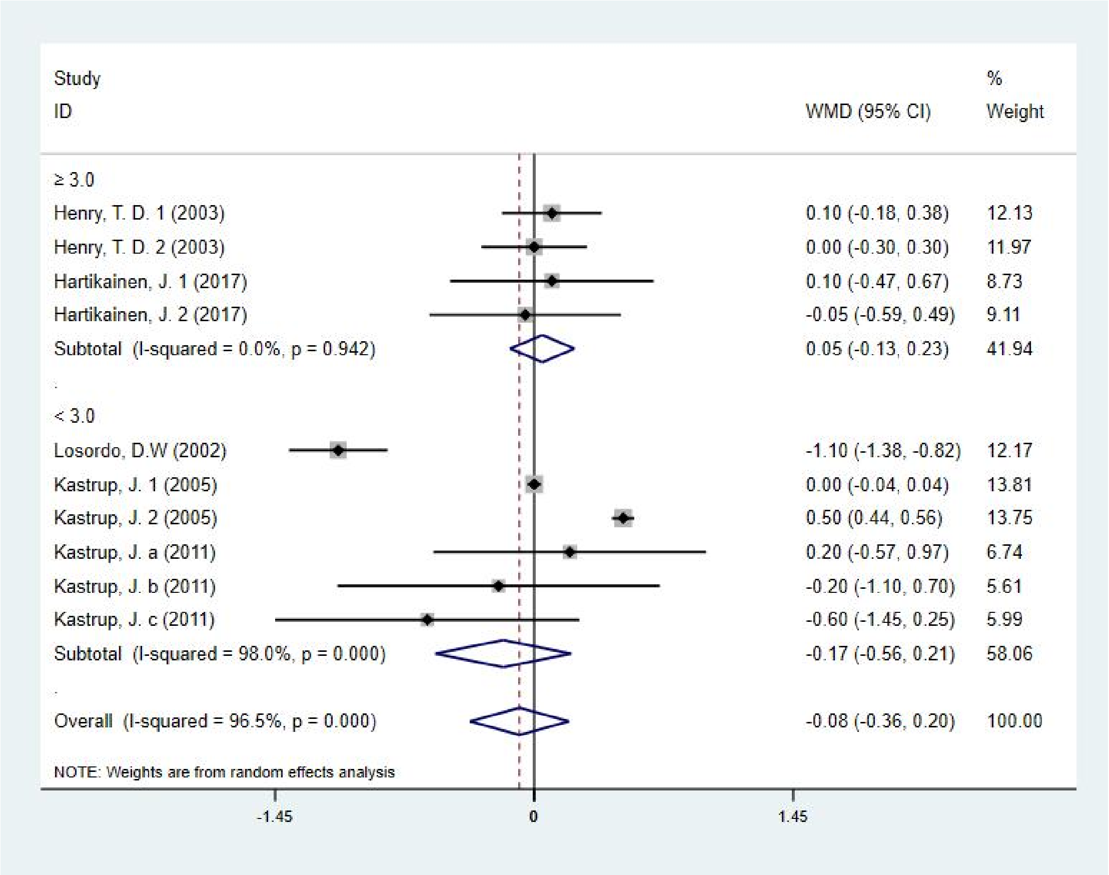

FIGURE S21 | Subgroup analysis of the effect of GF on CCS angina class based on Baseline CCS angina class. CCS: Canadian Cardiovascular Society; WMD: weighted mean difference; CI: confidence interval; ID: identification.

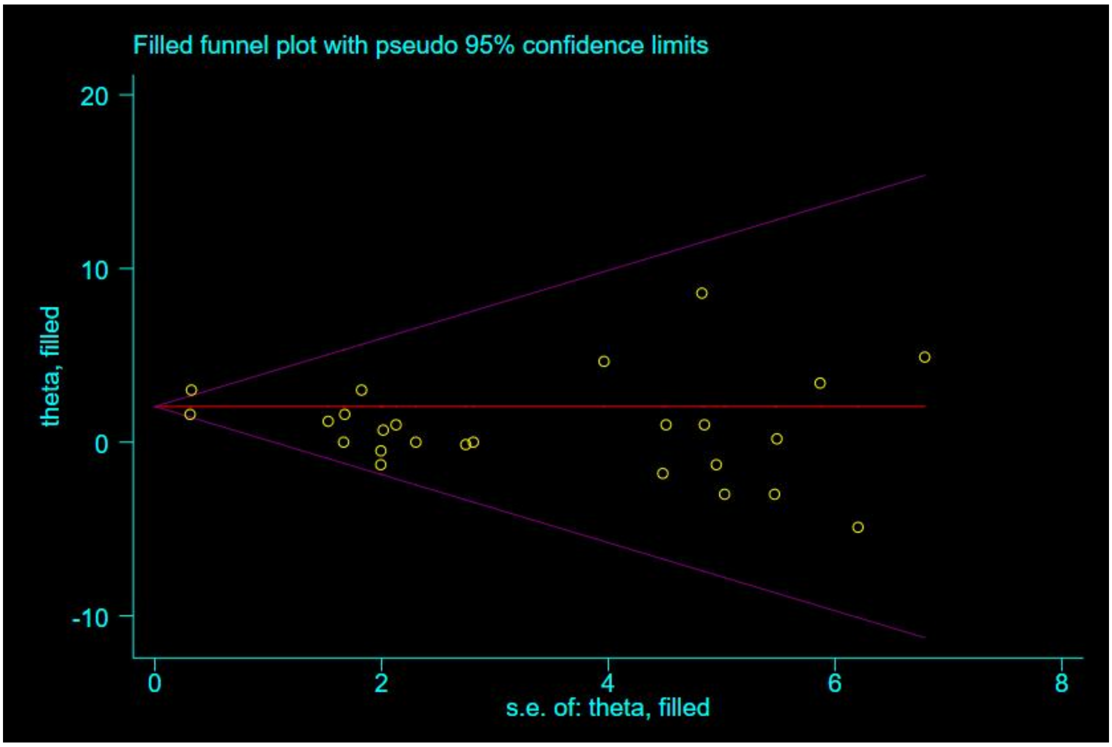

FIGURE S22 | Contour-enhanced funnel plots based on trim-and-fill method.
